# Characterization of immature ovarian teratomas through single-cell transcriptome

**DOI:** 10.3389/fimmu.2023.1131814

**Published:** 2023-03-03

**Authors:** Minyuan Cao, Yun Deng, Yiqi Deng, Jing Wu, Chongyi Yang, Zijun Wang, Qianqian Hou, Huancheng Fu, Zhixiang Ren, Xuyang Xia, Yue Li, Wei Wang, Heng Xu, Xin Liao, Yang Shu

**Affiliations:** ^1^ State Key Laboratory of Biotherapy and Cancer Center, West China Hospital, Sichuan University, Chengdu, Sichuan, China; ^2^ College of Life Sciences, Sichuan University, Chengdu, Sichuan, China; ^3^ Research Core Facility of West China Hospital, Sichuan University, Chengdu, Sichuan, China; ^4^ Department of Pathology, West China Second Hospital, Sichuan University, Chengdu, Sichuan, China; ^5^ Division of Laboratory Medicine, State Key Laboratory of Biotherapy, West China Hospital, Sichuan University, Chengdu, Sichuan, China; ^6^ Gastric Cancer Center, West China Hospital, Sichuan University, Chengdu, Sichuan, China

**Keywords:** immature teratoma, single cell RNA sequencing, pluripotent stem cell, immature neuron, radial glia, tumor immune microenvironment, germ cell tumor

## Abstract

**Introduction:**

Immature ovarian teratomas are a type of malignant germ cell tumor composed of complicated cell types and are characterized by pathological features of immature neuroectodermal tubules/rosettes. However, there is a lack of understanding of patient-derived immature ovarian teratomas (PDT) at the single cell level. Moreover, whether stem cell lines derived from immature teratomas (CDT) can be used as models for research on PDT remains to be elucidated.

**Methods:**

Single-cell RNA sequencing (scRNA-seq) and subsequent bioinformatic analysis was performed on three patient-derived immature ovarian teratomas (PDT) samples to reveal the heterogeneity, evolution trajectory, and cell communication within the tumor microenvironment of PDT. Validations were conducted in additional seven samples through multiplex immunofluorescence.

**Result:**

A total of qualified 22,153 cells were obtained and divided into 28 clusters, which can match to the scRNA-seq annotation of CDT as well as human fetal Cell Atlas, but with higher heterogeneity and more prolific cell-cell crosstalk. Radial glia cells (tagged by SOX2) and immature neuron (tagged by DCX) exhibited mutually exclusive expression and differentiated along distinct evolutionary trajectory from cycling neural progenitors. Proportions of these neuroectodermal cell subtypes may play important roles in PDT through contributing to the internal heterogeneity of PDTs. Moreover, the immune cells in PDTs were infiltrated rather than teratoma-derived, with more abundant macrophage in immature neuron than those in radial glia cells, and the infiltrated macrophage subtypes (i.e., M1 and M2) were significantly correlated to clinical grade. Overall, suppressed evolution process and transcriptome regulation in neuroectodermal cells, reduced cell-cell crosstalk, higher M1/M2 proportion ratio, and enhanced T cell effects in tumor microenvironment are enriched in patients with favorable prognosis.

**Discussion:**

This study provides a comprehensive profile of PDT at the single cell level, shedding light on the heterogeneity and evolution of neuroectodermal cells within PDTs and the role of immune cells within the tumor microenvironment. Also, our findings highlight the potential usage of CDTs as a model for research on PDT.

## Introduction

Human patient-derived immature teratoma (PDT) is a type of rare germ cell tumors that mostly affect young women. Immature teratomas are distinguished from mature teratomas in terms of both histological presentation of immature/embryonic tissue and clinically malignant behavior (1), and harbor frequent genetic homozygosity and a common cellular origin (2). The proportions of primitive immature neuroectodermal tissue present in PDTs are correlated with the tumor grade and prognosis (3). A majority of patients with grade 2/3 PDTs at stage I would experience a favorite prognosis after chemotherapy treatment (4, 5). However, some patients are at high risk of metastasis, particularly those with high grade and stage, requiring a better understanding to improve the therapeutic outcomes. Multiple studies has profiled the genomic and transcriptome of PDTs, revealing activation of telomerase (6), limited somatic mutations and copy number alterations (7–9), which cannot explain the high metastatic potential of some PDT cases. The possible reason would be the heterozygous and complex components of PDTs, and some cell types may contribute to the high malignant behavior, which cannot be identified at bulk-sequencing level. Recently, the development of single-cell RNA sequencing (scRNA-seq) has made it possible to comprehensively investigate the complex cell components and developmental trajectories. Despite that scRNA-seq has been conducted on a single pediatric PDT case (10), analysis on the heterogeneity and complexity of adult PDTs is far beyond to be elucidated.

On the other hand, injecting human pluripotent stem cell (hPSC) into immunodeficient mice, where the cells attach and differentiate in a semi-random fashion into all three germ layers, can thus generate cell-derived immature teratomas (CDTs) (11–13). Actually, CDTs are considered as a classical promising platform for modeling multi-lineage development (14), and have been systematically investigated through scRNA-seq with 23 teratomas from four hPSC cell lines, revealing around 20 cell types across three germ layers (15, 16). Given the immature status of CDTs, which can be sculpted through molecular operation (e.g., CRISPR-Cas9 knockout) (15), it would also be a potential model to study on the molecular basis and screen the druggable targets for malignant immature teratoma in human. Therefore, comparison between PDTs and CDTs at single cell level should be performed as the first step.

In this study, we aim to profile the heterogeneity of ovarian PDTs in adults through conducing scRNA-seq in three cases, and comparing the similarities and differences between PDTs and CDTs, particularly focusing on the abundance and characteristics immature neuron, as well as the prolific interactions among different cell components. Our findings not only firstly profiled the ovarian PDTs at single cell resolution, but also demonstrated that CDTs could be used to investigate neurogenesis in PDTs.

## Methods

### Patients

In this study, three individuals with grade 3 immature ovarian teratomas, as defined by the WHO (17), were enrolled (**Table 1**). Additionally, a validation cohort consisting of 7 patients across grades 1, 2 and 3 was included (**Supplementary Table 4**). This study was approved by the Institutional Review Board of West China Second University Hospital (IRB No. 2020112), and informed consent was obtained from patients (18).

**Table 1:** Clinical characteristics of three patients with immature ovarian teratoma.

Case	Grade	Age (years)	BMI	AFP (ng/ml)	Laterality	Tumor size	Surgery	Prognosis
PDT01	3	24	19.3	192.2	Left	12.7 × 5.7 × 6.5 cm	Tumor enucleation	EFS 2.5 years
PDT02	3	23	22.5	56.7	Left/Right	9.3 × 8.4 × 8.6 cm (Left); 0.8 × 0.7 × 0.8 cm (Right)	Tumor enucleation	EFS 2 years
PDT03	3	36	22.1	215.8	Left/Right	13.2 × 9.4 × 14.7 cm	Hysterectomy; USO	metastasis 2 years

EFS, Event Free Survival; AFP, α-Fetoprotein; USO, Unilateral Salpingo-Oophorectomy

### Sample collection and processing

Immature ovarian teratomas were dissected and sheared on ice after being washed with Hanks’ balanced salt solution (HBSS). The tissues were then digested for 1 hour at 37°C with collagenase I (2 mg/ml) (Gibco 1710-0017), collagenase IV (1 mg/ml) (Gibco 1710-4019), and 0.25% pancreatic enzymes (Gibco 25200-056). To remove cell debris and large clumps, the digested tissues were filtered through a 40-mm strainer. At 4°C, the suspensions were pelleted at 500g for 5 minutes. After lysis with 10 RBC Lysis Buffer (Thermo Fisher Scientific, 00-4300-54), the pellets were resuspended in HBSS with 0.04% bovine serum albumin (BSA) to determine cell viability and concentration (Counting Star, Aber Instruments Ltd.). The remaining cells were pelleted at 500g for 5 minutes at 4°C before being stored at 80°C. Finally, single-cell suspensions were diluted to about 5000 cells per milliliter. All tissues were obtained from West China Biobanks at Sichuan University’s West China Hospital’s Department of Clinical Research Management.

### Library preparation and sequencing

According to the directions provided by the manufacturer in the Chromium Single Cell 3’ Reagents Kits v2 User Guide, the Chromium Single Cell 3’ Library & Gel Bead Kit v2 (PN-120237), Chromium Single Cell 3’ Chip Kit v2 (PN-120236), and Chromium i7 Multiplex Kit (PN-120262) were utilized. Phosphate-buffered saline (PBS) + 0.04% BSA was used to wash the single-cell suspension twice. With the use of the TC20 Automated Cell Counter, cell quantity and concentration were verified. A 10x Genomics Chromium Controller machine was used to generate gel beads in emulsion (GEM) from the cells right away. A 10x Genomics Chromium Single Cell 3’ reagent kit (V2 chemistry) was used to prepare barcoded complementary DNAs (cDNAs), which were then recovered, purified, and amplified to provide enough for library construction. With the use of an Agilent Bioanalyzer 2100, library concentration and quality were evaluated. For PE150 sequencing, libraries were performed on Illumina’s NovaSeq 6000 platform.

### Single cell RNA-seq processing

Analysis on single cell transcriptome was performed with the pipeline as we described previously (19, 20). Briefly, the raw data was processed using Cell Ranger (v3.0) (21). Following Cell Ranger processing, a raw unique molecular identifier (UMI) count matrix was generated, which was then converted into a Seurat object by the R package Seurat (22). All Cell Ranger commands were executed with the default settings. Cells with UMI numbers below 500, gene numbers below 300 or greater than 6,000, or mitochondrial-derived UMI counts of more than 15% were considered low-quality and were removed. Furthermore, we used Scrublet (v0.2) to calculate doublet scores and predicted potential doublets for each cell, with expected doublet rate = 0.06 (23). The predicted doublets were then removed. After quality control, 6274, 8435, and 7444 cells were left for each patient for subsequent analysis.

### Seurat data integration

The Seurat v4 data integration pipeline was used to integrate the data (22). To summarize, we first used total-counts normalization to normalize the filtered counts matrix before log-transforming the result. We identified highly variable genes in each sample and chose the top 3000 genes that appeared to be overdispersed across the most teratomas. We then used sctransform (24) to normalize, scale, and correct for mitochondrial and ribosomal read percentages. Nonbiological batch effects were removed by performing a canonical correction analysis on individual samples using the Seurat functions FindIntegrationAnchors and IntegrateData.

### Cell types clustering and annotation

Following data integration, the RunPCA function was used to perform PCA, with the first 40 principal components being used. The RunUMAP function was used to generate UMAP. The Seurat functions FindNeighbors and FindClusters were used to find clusters with a resolution of 2.0. We then manually examined the marker genes for each cluster and annotated the cell type based on canonical marker expression (**Supplementary Table 2**).

### Cell type validation

Signature genes in human fetal Cell Atlas (25) and CTD teratomas (15) were defined as the top 50 DEGs with the lowest p values for each cell type. The signature scores for each cell type were then calculated using Seurat’s AddModuleScore function. The signature scores that resulted were interpreted as correlation assessments.

### Cell population purity

In this study, we compared the heterogeneity of CDT and PDT cell types using ROGUE (26), an entropy-based universal metric for assessing the purity of single cell populations. The ROGUE index has been scaled from zero to one. One represents a completely pure subtype with no significant genes, and zero represents the population’s most heterogeneous state.

### PCA based transcriptome heterogeneity

For PCA reduction projection, expression matrix from PDT and CDT was extracted from SCT assay in Seurat objects. To reduce batch effects, we only select shared genes across PDT and CDT, and their expression matrix were normalized as Z-scores. PCA embedding were calculated on combined expression matrix by R prcomp function. In CDTs, the PCA similarity distance was calculated as the Euclidean distance between each CDT sample to the lower left CDT sample using the Pythagorean theorem.

### Trajectory analysis

Monocle 2 was used to perform pseudotime analysis to determine the dramatic developmental trajectory and translational relationships of neural cell types (27). 1500 significantly changed features were identified by the FindVariableFeatures function in Seurat with the vst method and used for cell ordering. The default parameters were used to calculate CytoTRACE scores, and the results were used to infer the origin of neural cells (28).

BEAM analysis (q-value < 1e-50) was used to identify cell differentiation-related genes, which were then visualized using the plot genes branched heatmap function.

### SCENIC analysis

SCENIC analysis was carried out as previously described (29). The pySCENIC package (version 0.10.3) was used, which is a lightning-fast Python implementation of the SCENIC pipeline. The Wilcoxon rank sum test was used to identify the differentially activated TFs of each subcluster in comparison to all other cells of the same cell type in PDTs.

### Define myeloid and T cells related phenotypes

The M1/M2 phenotype of each macrophage cell was defined as the mean expression of gene signatures (30). M1 macrophage signature includes IL23, TNF, CXCL9, CXCL10, CXCL11, CD86, IL1A, IL1B, IL6, CCL5, IRF5, IRF1, CD40, IDO1, KYNU, and CCR7. the M2 macrophage signature includes IL4R, CCL4, CCL13, CCL20, CCL17, CCL18, CCL22, CCL24, LYVE1, VEGFA, VEGFB, VEGFC, VEGFD, EGF, CTSA, CTSB, CTSC, CTSD, TGFB1, TGFB2, TGFB3, MMP14, MMP19, MMP9, CLEC7A, WNT7B, FASL, TNFSF12, TNFSF8, CD276, VTCN1, MSR1, FN1 and IRF4.

Phagocytosis genes contain MRC1, CD163, MERTK, and C1QB (30). Antitumor cytokines include TNF, IFNB1, IFNA2, CCL3, TNFSF10, and IL2 (31). Protumor cytokines contain IL4, IL5, IL13, IL10, GATA3 and CCR4 (31). Angiogenesis genes include VEGFA, VEGFB, VEGFC, PDGFC, CXCL8, CXCR2, FLT1, PGF, CXCL5, KDR, ANGPT1, ANGPT2, TEK, VWF, and CDH5 (30). The Effector T cells traffic phenotype was defined as the mean expression of CXCL9, CXCL10, CXCL11, CX3CL1, CCL3, CCL4, CX3CR1, CCL5, and CXCR3 (31). T cell cytotoxic signature contains GZMA, GZMB, GZMK, IFNG, NKG7, PRF1, CST7, CCL4 and TYROBP (32, 33). T exhaustion signature contains PDCD1, LAG3, TIGIT, HAVCR2 and CTLA4. The corresponding mouse signature were defined by their human homologous genes by function convert_mouse_to_human_symbols in R package nichenetr.

### Cell-cell interactions analysis

The quantification of ligand-receptor pairs among different cell types was used to assess cell-cell communication. CellphoneDB or CellChat (34, 35) software was fed gene expression matrices and metadata with cell type annotations. CellPhoneDB’s default database and parameters were used. PDT02 and PDT01/03 were analyzed separately for CellChat, and the netVisual_diffInteraction function was used to visualize the differential interaction strength between the two groups by using cycling neural progenitors as target cells.

### RNA velocity analysis

The velocyto CLI (36, 37) was used to count the spliced and unspliced UMIs for each gene in each cell, and scvelo (37) was used to perform the subsequent analyses. Specifically, the function scv.pp.moments was used to compute the moments for each cell with the default parameters of normalized spliced/unspliced counts. These moments aided RNA velocity estimation in the function scv.tl.velocity, with the mode set to ‘stochastic’. Using the function scvelo.tl.velocity graph, a velocity graph representing transition probabilities was constructed based on estimated velocities. The velocity graph was then used to embed RNA velocities in the UMAP as streamlines using the scv.pl.velocity embedding stream function.

### Multiplexed immunofluorescence staining

All involved surgical tumor tissues from 7 additional PDT patients were processed into paraffin blocks. Multiplex mIF staining was performed as our previous described (19), using the Opal 7-color kit (Akoya Bioscience, NEL801001KT). Tissues were sliced into 4μm sections and heat in the retrieval solution of citric acid or EDTA to recover antigens. All the relative antibodies, CD163 (ab182422, Abcam, 1:500, Opal 620), CD68 (ab269587, Abcam, 1:1000, Opal 570), SOX2 (11064-1-AP, Proteintech, 1:100, Opal 690), Doublecortin (ab18723, Abcam, 1:1000, Opal 520), were evaluated *via* immunohistochemistry. Briefly, the sections were dewaxed with xylene and ethanol was used for rehydration. Microwave treatment was performed for antigen retrieval with buffer (pH 9.0 or 6.0). Next, all sections were blocked using an antibody diluent/block (72424205; Akoya Bioscience), Slides were incubated with a primary antibody followed by secondary reagents and tyramide signal amplification reagents at room temperature for 10 min (Opal 480, Opal520, Opal 620, and Opal 690, Akoya Bioscience, 1:100). MWT antigen retrieval was performed until all markers were stained. Finally, DAPI-based nuclear staining was performed at room temperature for 5 min. Then the sections were mounted using anti-Fade fluorescence mounting medium (ab104135, Abcam), Samples were scanned by Vectra Polaris Automated Quantitative Pathology Imaging System and analyses were performed with QuPath software.

## Results

### Single-cell profiling of human patient-derived immature ovarian teratomas

**Figure 1:**
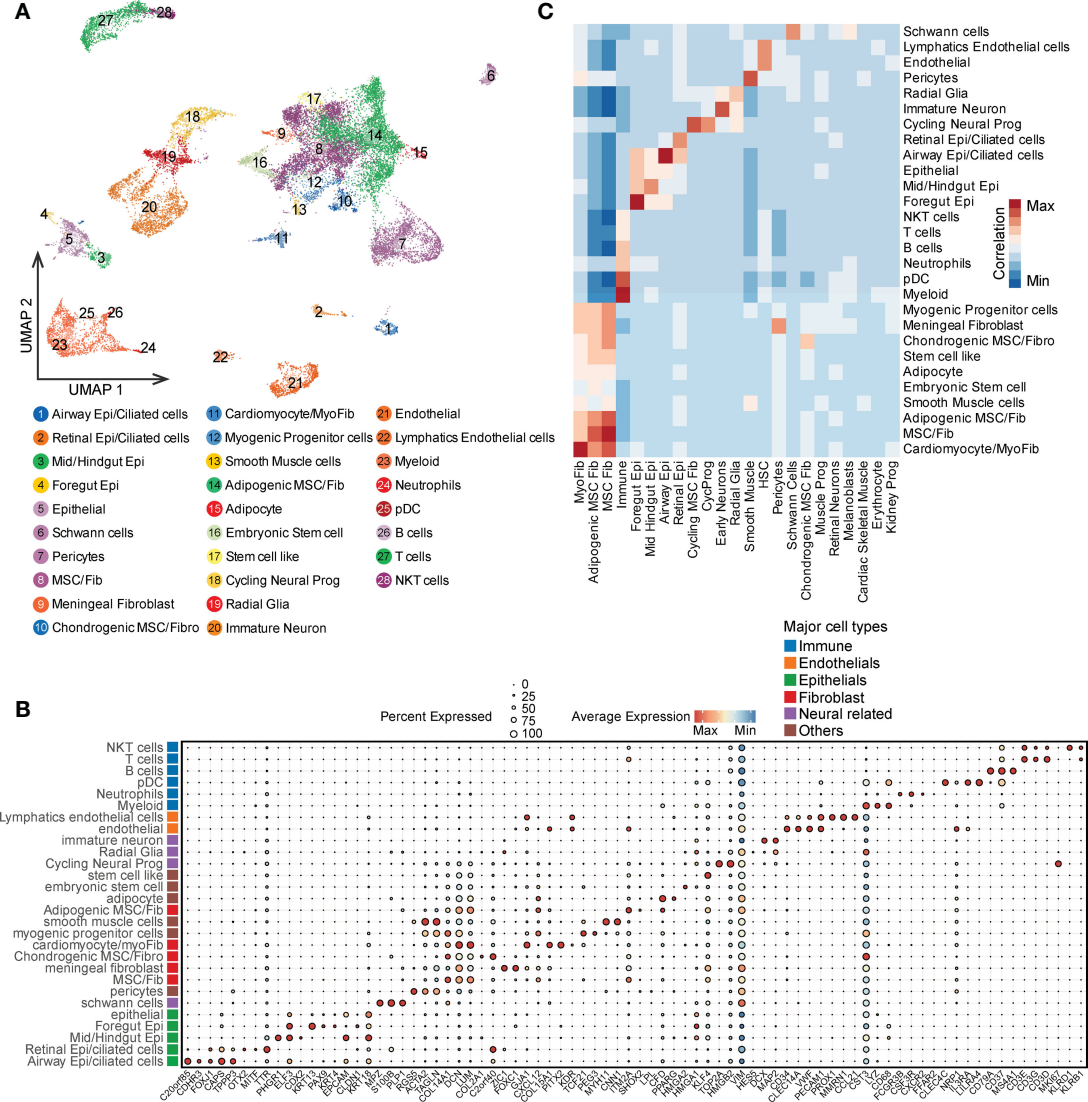
Single cell profile of ovarian immature teratomas. **(A)** UMAP visualization of cell types identified from scRNA-seq of the three patients with immature ovarian teratoma. **(B)** Canonical markers of each cell types in PDT visualized by bubble plot. **(C)** Correlation of the average expression of each PDT cell type with that of each H1 derived CDT cell type.

To explore the cellular composition of teratoma, we generated single-cell gene expression matrices of clinically annotated immature ovarian teratomas from 3 patients (**Table 1** and **Supplementary Figure 1**). After quality control, we acquired transcriptomes of 22,153 cells, which can be divided into 28 clusters based on cluster-specific markers obtained by gene differential expression analysis, including immune cells, epithelial cells, fibroblasts, and neuron cells (**Figure 1A** and **Supplementary Table 1**). These clusters were refined and manually annotated using canonical cell type markers, such as meningeal fibroblast (*ZIC1*, *CXCL12*), schwann cells (*MPZ*, *PLP1*), chondrogenic fibroblast (*COL2A1*, *SOX9*), and immature neuron (*DCX*, *MAP2*) (**Figures 1A, B** and **Supplementary Table 2**). Also, the expression of canonical marker genes was visualized for each cell type to assess the robustness of cell type annotations (**Supplementary Figure 2A**). To further validate the cell type annotations, we assessed the relationship between the expression signature of each teratoma cell type and that of the human fetal cell atlas (25), revealing that each teratoma cell type generally correlates with at least one human fetal cell type (**Supplementary Figure 2B**). For instance, immature neurons from the teratoma correlate with fetal human intestine neurons, airway epi/ciliated cells correlate with human fetal lung ciliated epithelial cells, whereas pericytes present at intervals along the walls of capillaries and contain contractile fibers as vascular smooth muscle thus correlate accordingly (38). Such correlation confirmed the similarity between different components of teratomas and that of fetal cells.

Moreover, given injection of hPSC into immunodeficient mice can generate cell-derived immature teratomas (CDTs), which has been profiled through scRNA-seq (**Supplementary Table 3**) (15), we thus compared the expression signatures of their correlated cell types with PDTs (**Supplementary Figure 2C**). Not surprisingly, most of the cell types in PDTs are strongly correlated with the expected cell types in CDTs, while some discrepancies were observed possibly induced by the differences in developmental stage and xenograft-specific expression (**Figure 1C**).

### Similarities and differences between PDTs and CDTs

**Figure 2:**
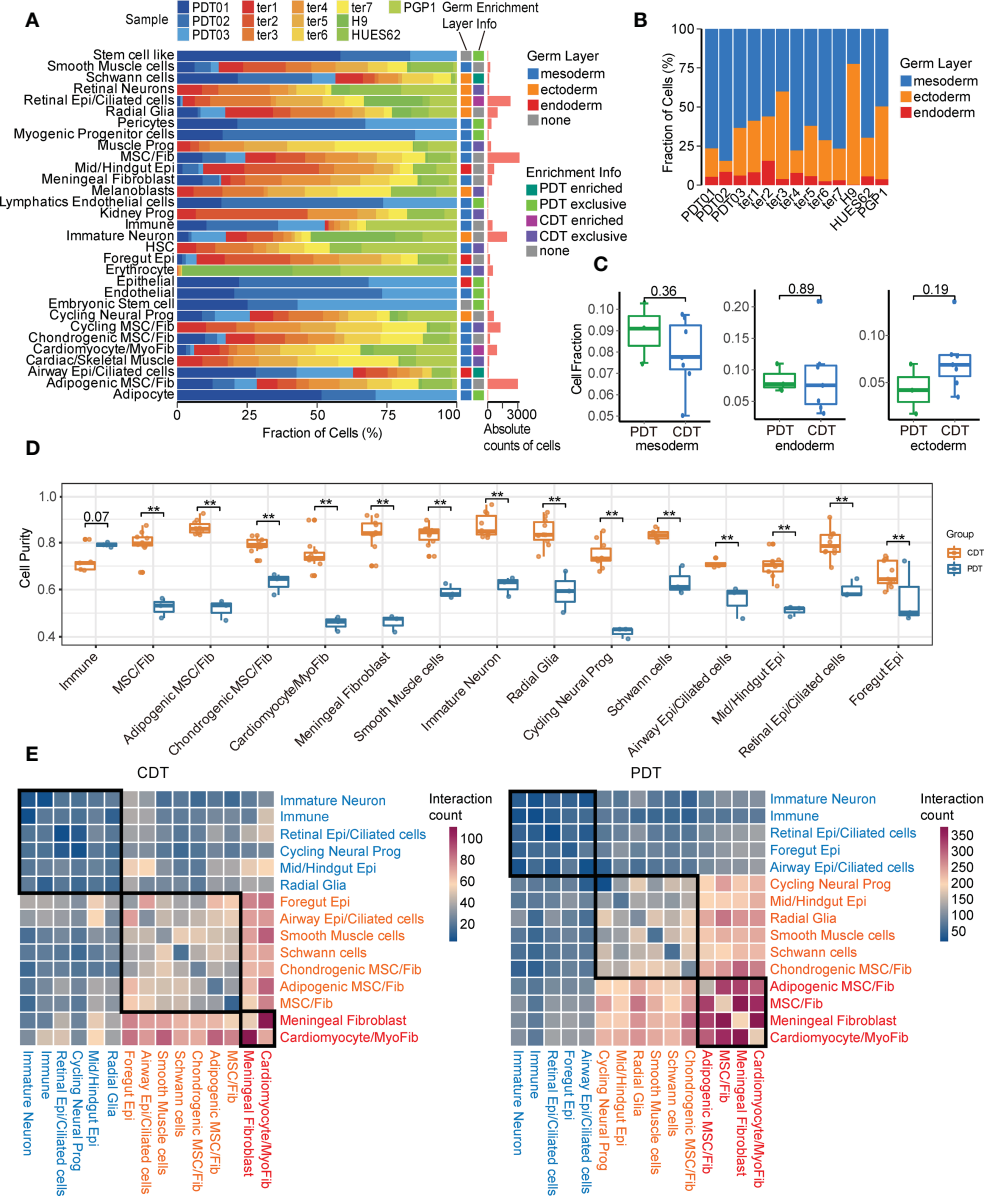
Overall comparison of PDT and CDT in all single cell clusters. **(A)** Proportion of cells from PDTs and CDTs present in each cell type. **(B)** Proportion of germ layer origin in each teratoma. **(C)** Proportion disparity between PDT and CDT across all three germ layers. **(D)** Cell purity (calculated by ROGUE) of shared cell types in PDT and CDT. **(E)** Cell-cell interaction network heatmap of 15 shared cell types in PDT and CDT. Text in red, orange, and blue indicated cell types belong to high, medium, and low interaction module, respectively. ** P<0.01

To further investigate the similarities and differences between PDTs and CDTs, we first compared the proportions of 31 cell types. Around half of the cell types (15 out of 31) were shared by both teratoma types (e.g., radial glia, immature neuron, and schwann cells), whereas eight were distinct in PDTs (e.g., embryonic stem cells, endothelial cells and pericytes) and eight were distinct in CDTs (e.g., retinal neurons, malenoblasts, and cardiac/skeletal muscle) (**Figure 2A**). Considering the germ layer, most of the cells from both PDTs and CDTs are derived from mesoderm and ectoderm but less from endoderm (**Figure 2B**), suggests that endoderm is less prevalent during early development. Despite distinct cell types observed in PDTs and CDTs, there is no statistically significant difference between these two teratomas types in terms of germ layer components (**Figure 2C**). We next compared the heterogeneity between PDTs and CDTs in their shared 15 cell types and found that all cell types but not immune cell from PDTs have significant lower purity than that from CDTs (**Figure 2D**), suggesting the heterogeneity and complexity of PDTs. Moreover, cell-cell interaction network of the shared cell types was also estimated and compared between PDTs and CDTs, revealing higher overall interactions among PDT-derived cell types (**Figure 2E**). Three interaction modules (e.g., high, medium, and low) were defined based on the cell-cell interaction counts in both PDTs and CDTs (**Figure 2E**). Interactions between all five fibroblast cell types and other cell types are found in high/medium modules, particularly meningeal fibroblast and cardiomyocyte/myofibroblast are in high modules in both teratoma types, whereas interactions between all four epithelial cell types and other cell types are found in low/medium module, particularly retinal epithelial are in low modules in both teratoma types (**Figure 2E**). For neuroectodermal cells, immature neuron exhibited the least cell-cell interactions with all the rest cell types, whereas cycling neural progenitor and radial glia cells increase the intensity of their interactions from low in CDTs to medium in PDTs (**Figure 2E**), suggesting more activated neural development in malignant teratomas.

### Immature neuron abundance related transcriptome heterogeneity in teratomas

**Figure 3:**
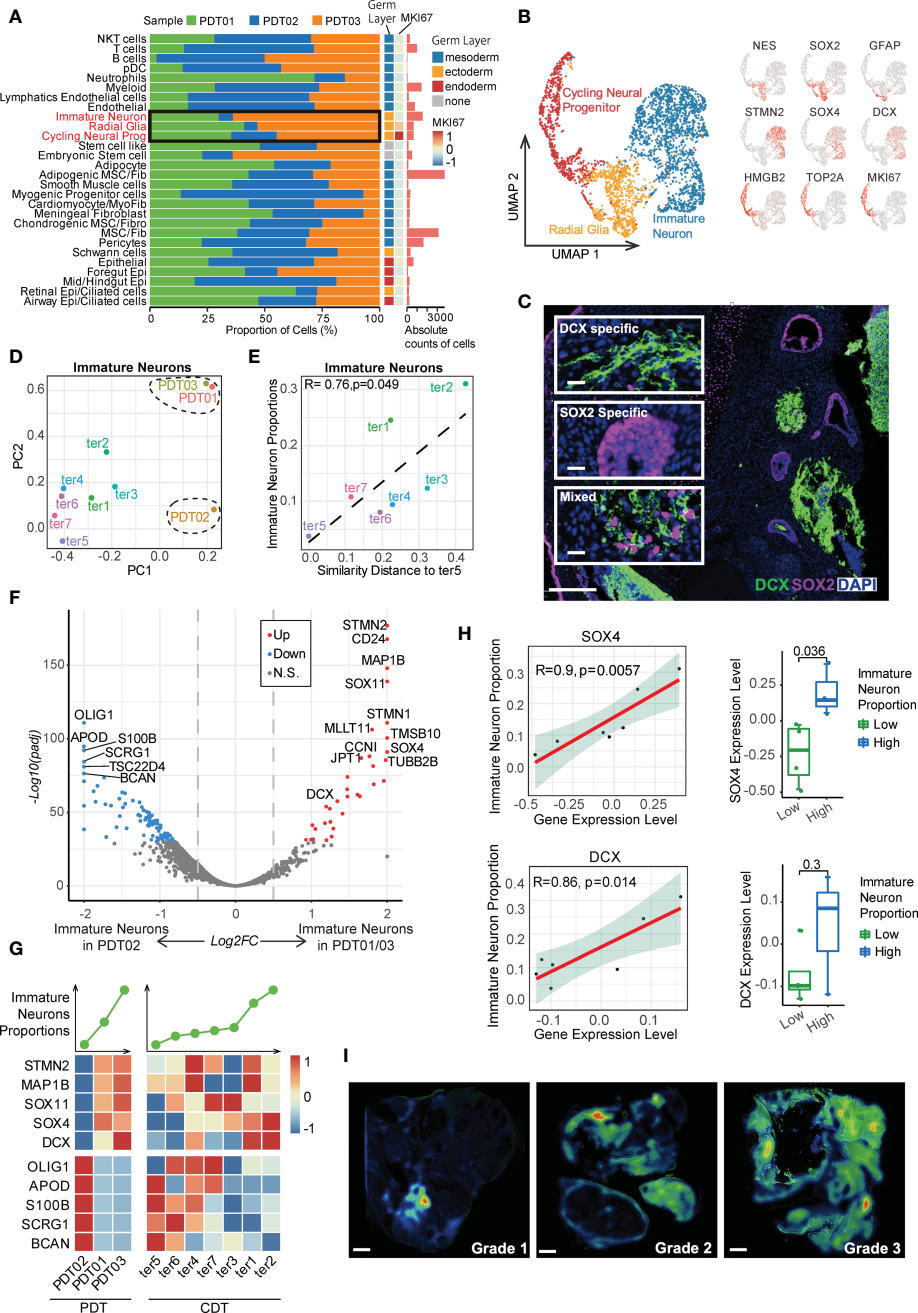
Characteristics of neuroectodermal cell types of PDT and CDT. **(A)** The proportion of cells from three PDT samples across all cell types. **(B)** Left: UMAP presentation of three main neuroectodermal cell types in PDT; Right: expression levels of their representative markers. **(C)** mIF staining with antibodies against DCX (green) and SOX2 (red). **(D)** Immature neuron transcriptome heterogeneity illustrated by PCA projection across all teratomas. **(E)** The correlation between PCA similarity distance to ter5 and immature neuron proportions in H1-derived CDTs. **(F)** DEGs between PDT02 and PDT01/03 in immature neurons illustrated by volcano plot. **(G)** Top: line plot to reveal the immature neuron proportion across all samples in PDT and CDT; Bottom: expression level heatmaps of top DEGs in immature neurons. **(H)** The correlation between gene expression level and immature neuron proportions with linear and categorical model. **(I)** Average optical density (AOD) of DCX antibody in immunofluorescence.

Given immature and embryonic tissue from all three germ layers can be found in immature teratomas, and the amount of primitive neuroectodermal tissue is highly predictive of prognosis (3, 39), we first focused on the neuroectodermal cell types. In this study, PDT02 had a lower proportion of ectodermal cells than the other two patients (**Figure 3A** and **Supplementary Figure 3A**), consistent with her event-free survival status and low AFP level (**Table 1**). Three major neuroectodermal cell types were characterized by specific markers (**Supplementary Table 2**), including radial glia (*SOX2*, *NES*), cycling neural progenitor (*HMGB2*, *MKI67*), and immature neuron (*STMN2*, *DCX*) (**Figure 3B**), which is perfectly matched to the cell types with the same marker genes in CDTs (**Supplementary Figure 3B**). As validation by multiplexed immunofluorescence (mIF) staining, *DCX^+^* and *SOX2^+^* cells were either present in *DCX*-/*SOX2*-specific region or expressed in the mixed region in mutually exclusive manner, and exhibited different morphological features (**Figure 3C** and **Supplementary Figure 3C**). According to the guideline of gynecologic pathology and WHO-defined histopathology, immature teratomas are characterized by immature neuroepithelium (rosettes and tubules), and neuroectodermal elements can be highlighted by neural markers, including *NSE*, *GFAP*, and *SOX2*, among them, *SOX2* is more specific for immature neural tissue and diagnosis of immature teratoma (4). Therefore, enriched presentation of these genes indicated the dominate role of radial glia to determine immature teratoma.

Next, we performed PCA analysis to estimate the transcriptional similarity of neuroectodermal cell types and found that all three PDTs were clustered together based on the transcriptome profile of both radial glia and cycling neural progenitor, whereas immature neuron from PDT02 shared less similarity with that from PDT01/PDT03 (**Figure 3D** and **Supplementary Figure 3D**), suggesting the inter-heterogeneity among PDTs can be featured by immature neuron cells. Moreover, significant association of transcriptome disparity in H1-based CDTs (i.e., distance between ter5 and other CDTs in PCA) with the cell proportions was observed in immature neuron but not in radial glia and cycling neural progenitor clusters (**Figures 3D, E** and **Supplementary Figure 3E**). Next, we screened the differentially expressed genes (DEGs) of immature neuron cells between PDT02 and PDT01/PDT03, and found that a series of the top up-regulated genes (e.g., *STMN2*, *STMN1*, *CD24*, *SOX11*, *SOX4*, and *DCX*) were canonical markers for immature neurons and contribute to neuronal differentiation and migration during neurogenesis (**Figure 3F**) (40–44). For instance, *SOX4* and *SOX11*, which encode two neurogenesis-related transcription factors, are functionally essential for hippocampal neurogenesis (42). Consistently, some of these genes exhibited similar positive correlations between their expression levels and immature neuron proportions in CDTs (**Figure 3G**), with *SOX4* and *DCX* reached statistical significance in terms of linear and/or categorical approaches (**Figure 3H**). To further validate the findings, we performed mIF staining of DCX in validation cohort (n = 7) (**Supplementary Table 4**), and identified the positive correlations between the overall expression levels of DCX and teratoma grade (**Figure 3I** and **Supplementary Figure 3F**). However, due to the limited number of patients enrolled in the validation cohort, the correlation did not reach statistical significance (**Supplementary Figure 3F**). On the other hand, many top down-regulated genes are related to gliogenesis and glial differentiation (e.g., *OLIG1*, *OLIG2*, *APOD*, and *S100B*) (45) (**Figures 3F, G**), while expression of *S100B* and *SCRG1* exhibited negative correlation with immature neuron proportions in CDTs (**Supplementary Figure 3G**). In contrast, no trend has been observed for the correlation between markers gene expressions and the cell proportions in radial glia and cycling neural progenitor (**Supplementary Figures 3H, I**).

### Evolutionary trajectory analysis of major neuroectodermal cells in PDTs

**Figure 4:**
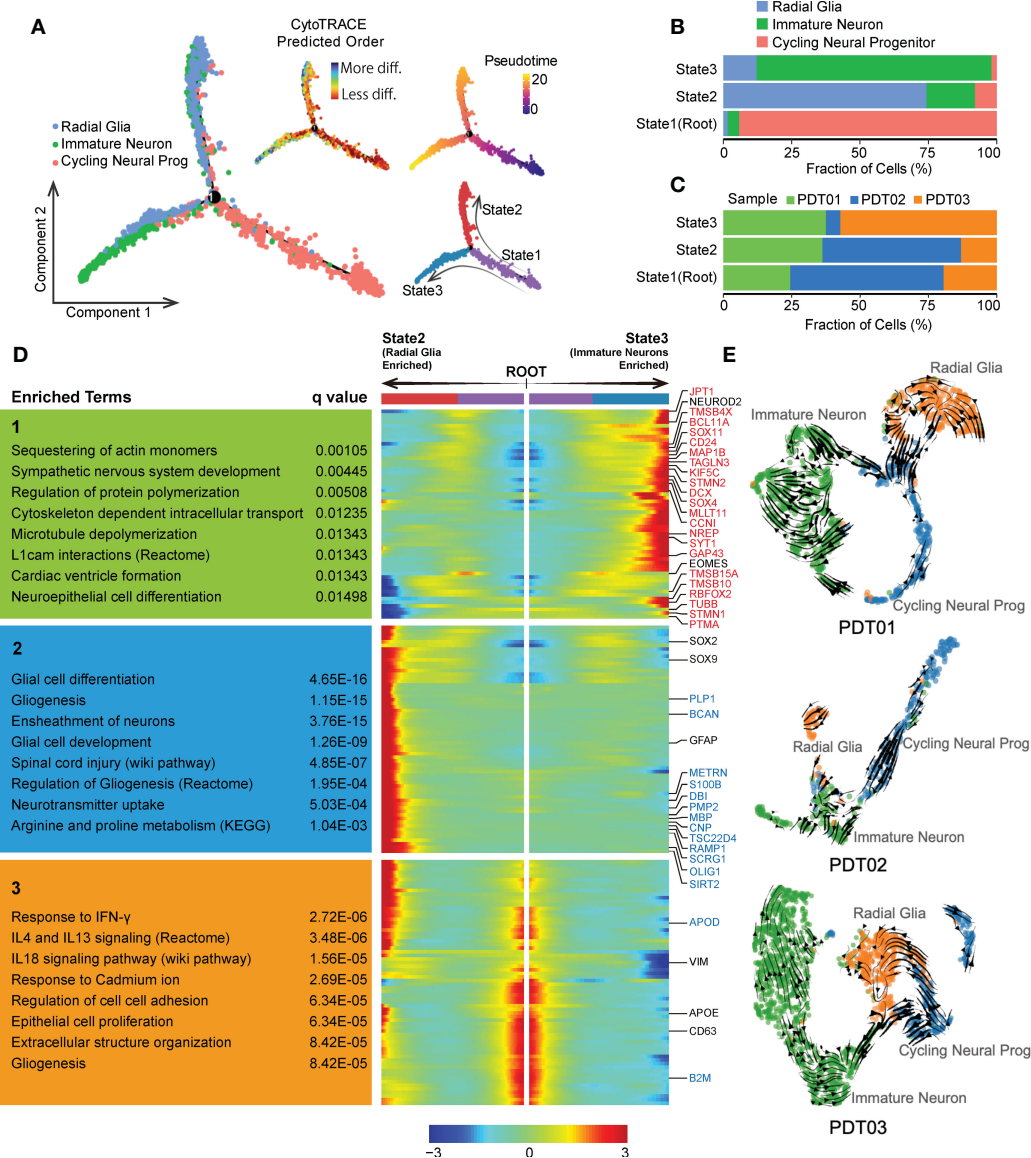
Evolution of neuroectodermal cells in PDT. **(A)** Monocle trajectory of three main neuroectodermal cell types, root was defined by state with minimum CytoTRACE score (less differentiated). **(B)** The proportion of three main neuroectodermal cell types across three monocle trajectory states. **(C)** Bar plot to reveal the proportion of three PDT samples across three monocle trajectory states. **(D)** Fluctuation of genes expression along biforked trajectories, genes in red and blue indicate up-regulated and down-regulated DEGs in **Figure 3E**, respectively. **(E)** UMAP-based scRNA velocities projections of three main neuroectodermal cell types across three PDT samples.

Given that neurogenesis process in teratomas could be critical to patients’ prognosis, we conducted Monocle-based cell trajectory analysis to investigate the evolutionary transitions of three neuroectodermal cell types described above. Two distinct differentiated paths were revealed along with the evolutionary trajectory from cycling neural progenitors to radial glia/immature neuron, enhancing the definition of three states (**Figure 4A**), including State1 (cycling neural progenitors dominant, defined as root), State2 (radial glia dominant) and State3 (immature neuron dominant) (**Figure 4B**). Similar trends were observed in CDTs (**Supplementary Figures 4A, B**). Neuroectodermal cells from PDT01/PDT02 are more enriched in State1/State2 compared with PDT03 (**Figure 4C**), which may contribute to the metastatic ability of PDT03 according to our clinical follow-up (**Table 1**). Finally, tracing the gene fluctuation along biforked trajectories, genes that differ as a function of pseudotime were identified as incrementally up-regulated or down-regulated from root to State2 or State3, and can be grouped into three clusters (Cluster1, Cluster2 and Cluster3). Genes in Cluster1 were highly expressed by immature neurons and associated with nervous system development and neuron actin remodeling (**Figure 4D**). Interestingly, many of them are up-regulated genes identified in comparison between PDT01/PDT03 and PDT02 (**Figures 3F**, and **4D**). Genes in Cluster2 were up-regulated in radial glia cells, consisting of many down-regulated genes described above and enriched in several neuron development related pathways, including glial cell differentiation (**Figures 3F**, and **4D**). Finally, genes in Cluster3 were up-regulated in cycling neural progenitors and were enriched in chemokine and cytokine signaling regulation (**Figure 4D**).

We also conducted RNA velocity-based trajectory inference to delineate cellular differentiation of neuroectodermal cells. Consistently, cycling neural progenitors were identified as the origin of the global differentiation processes during neuroectoderm diversification to functional neural subtypes (**Figure 4E**). Moreover, samples from PDT02 exhibited suppressed neural cells differentiation from cycling neural progenitor to immature neuron/radial glia (**Figure 4E**), which may contribute to the lower abundance of immature neuron/radial glia in PDT02 than that in PDT01/PDT03.

### Transcriptional regulation and cell-cell crosstalk among tumor microenvironment in teratomas

**Figure 5:**
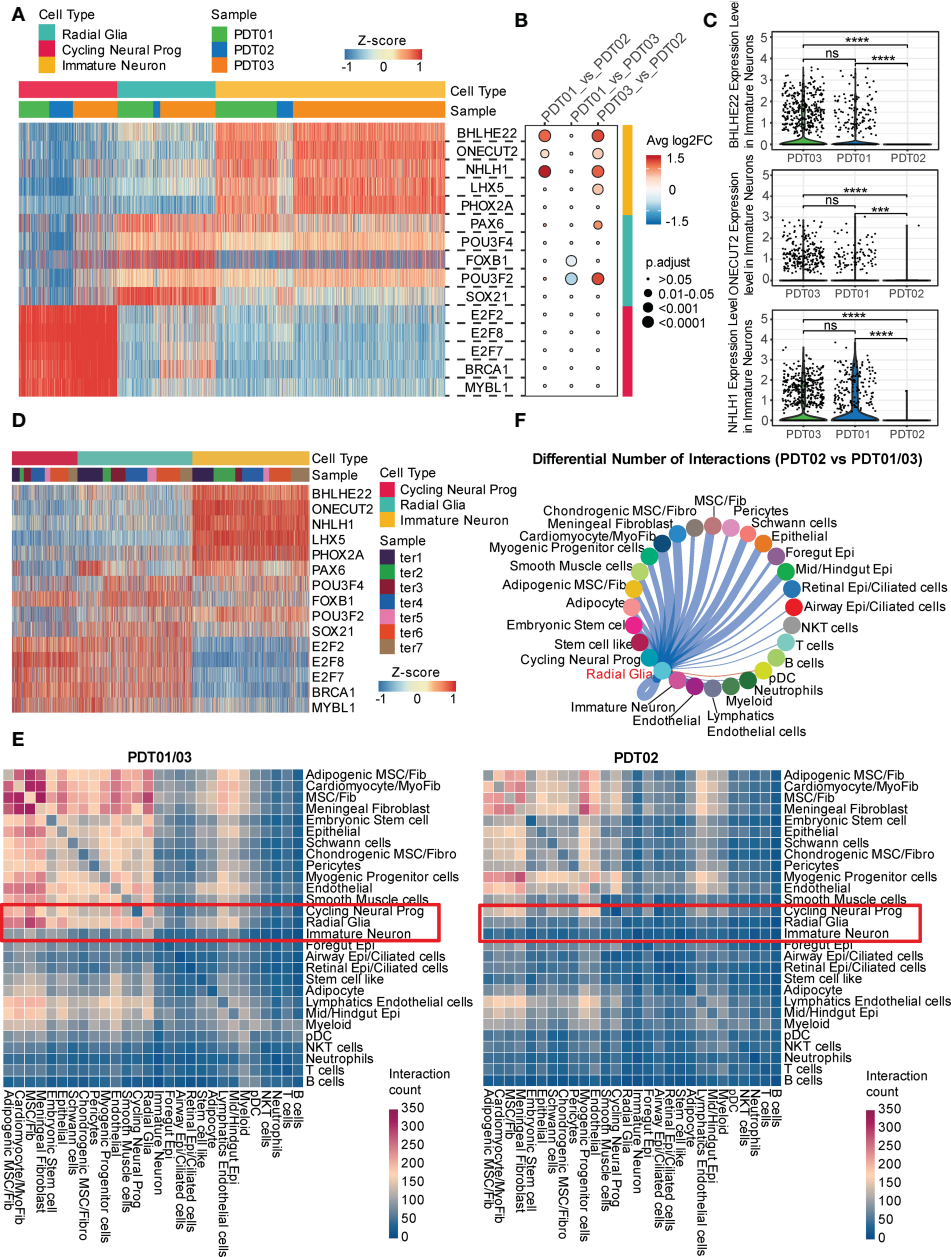
Transcriptional regulation and cell-cell crosstalk of PDT and CDT. **(A)** Area under the curve (AUC) scores of regulons estimated per cell in PDT by SCENIC. **(B)** The changes of regulon expression level between each paired of PDT samples in corresponding neural cell types. **(C)** The expression level of three immature neuron specific regulons across three PDT samples. **(D)** The area under the curve (AUC) scores of the same regulons as in **(A)** for each cell in CDT. **(E)** CellphoneDB-based heatmap of cell-cell interaction count across cell types in PDT01/03 and PDT02. **(F)** Inferred PDT02 *vs.* PDT01/03 differential number of interactions between radial glia and other cell types. *** P<0.001, **** P<0.0001. ns, stands for not statistically significant.

To elucidate transcriptional states across different cell types, SCENIC was performed to identify potential regulatory elements (**Supplementary Figure 5** and **Supplementary Table 5**). For neuroectodermal cell types, *BHLHE22*, *ONECT22*, and *NHLH1* are the specific regulons in immature neurons (**Figure 5A**), consistent with their reported role in cell-type determination, proliferation, and differentiation within the developing nervous system (46, 47). Interestingly, only the top transcription factors enriched in immature neuron are present at a significant lower level in PDT02 than PDT01/PDT03 (**Figures 5B, C**), suggesting the relative inactivation status of neuroectodermal cell in PDT02. Consistently, the top transcription factors are specifically over-presented in immature neuron of H1-derived CDTs (**Figure 5D**), but fail to correlate with the immature neuron proportions.

Next, we investigated the cell-cell crosstalk among different cell types through CellphoneDB and found more prolific overall interactions in PDT01/PDT03 than those in PDT02 (**Figure 5E** and **Supplementary Figure 6A**). For neuroectodermal cell types, radial glia has much fewer interactions with fibroblasts/epithelial cell types in PDT02 than those in PDT01/PDT03, whereas the differences were less pronounced in cycling neural progenitor and immature neuron (**Figure 5E**). To focus on the potential ligands that drive the transcriptome difference in three PDTs, we performed Cellchat to estimate the number of ligand-receptor interactions between neuroectodermal cells and other cell types. Consistent with the observations in CellphoneDB, PDT02 showed decreased number of interactions with primarily fibroblasts and epithelial cells in all three neuroectodermal cell types (**Figure 5F** and **Supplementary Figure 6B**), which might contribute to the better prognosis in PDT02.

### Tumor immune microenvironment heterogeneity in teratomas

**Figure 6:**
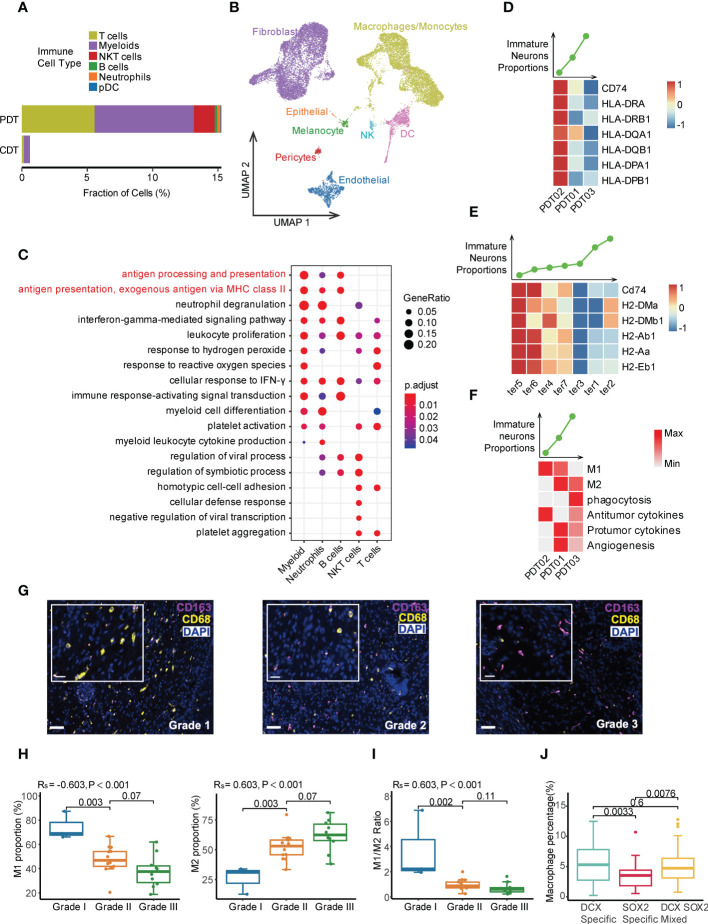
Tumor immune microenvironment heterogeneity in PDT and CDT. **(A)** The immune cell type proportions across PDT and CDT. **(B)** Cell types of mouse origin identified from scRNA-seq of H1-derived CDTs. **(C)** DEG enrichment of immune cells between PDT02 and PDT01/03. **(D)** Top: the immature neuron proportion across three PDT samples; Bottom: the expression heatmap of MHC-II components in myeloid cells. **(E)** Top: the immature neuron proportion across CDT samples; Bottom: the expression heatmap of MHC-II components in infiltrating mouse myeloid cells. **(F)** Top: the immature neuron proportion across three PDT samples; Bottom: the expression heatmap of myeloid related gene signatures in myeloid cells. **(G)** mIF staining with antibodies against CD163 (red) and CD68 (yellow). **(H)** Proportions of M1 and M2 macrophages across all grades of immature ovarian teratomas. **(I)** M1/M2 ratio across all grades of immature ovarian teratomas. Rs: spearman correlation. **(J)** Proportions of macrophages across DCX-/SOX2-specific and mixed regions.

Large differences were observed in terms of the cell type and proportion of immune cells between PDTs and CDTs. For instance, myeloid cells were dominantly identified in CDTs, compared with multiple immune cell types (e.g., myeloid, T cells, NK cells, B cells, and neutrophils) identified in PDTs (**Figures 1A**, **6A**, and **Supplementary Figure 2C**), while the immune cells exhibited distinct copy number variation (CNV) patterns compared to other cells across all PDT samples (**Supplementary Figure 7**), suggesting that most of the immune cells in PDTs were infiltrated rather than teratoma-derived. Additionally, immune cells were the only cell type that exhibited no difference between CDT and PDT in terms of cell purity (**Figure 2D**), further suggesting the altered origin of the immune cells in PDT. Because CDTs were generated in mice, we examined the mouse-derived cells from the scRNA-seq data of CDTs, and identified a large number of immune cells (mostly macrophage and dendritic cells because the mice are immunodeficient) (**Figure 6B**). Given myeloid is the most abundant cell types in both PDTs and CDTs, as well as the reported role of macrophages in aiding the initiation and progression of teratomas (48), we compared the expression profile of myeloid cells between PDT02 and PDT01/03, the DEGs were enriched in multiple immune related pathways, including major histocompatibility complex class II (MHC II) signaling (**Figure 6C**), compared to non-immune related pathways enriched in other cell types (**Supplementary Figure 8A**). Expression of multiple MHC-II components encoding genes were upregulated in myeloid cells from PDT02 compared to PDT01/PDT03, and the correlation of their expression with immature neuron proportion is in line with that of infiltrated myeloid cells of CDTs (**Figures 6D, E**), but not myeloid cells derived from CDTs (**Supplementary Figure 8B**), thus further confirmed the potential role of infiltrating immune cells in teratomas progression. Furthermore, we determined the gene signature based myeloid functional scores in each PDT, we found that PDT02, which has a lower proportion of immature neurons, showed an enrichment of M1 polarization and antitumor cytokine scores (**Figure 6F**). Conversely, PDT01/PDT03, with a higher proportion of immature neurons, showed an enrichment of M2 polarization, phagocytosis, protumor cytokine, and angiogenesis scores (**Figure 6F**). This was further confirmed by mIF staining in the validation cohort, which showed a positive association between the percentage of M2 macrophages and teratoma grading, whereas a negative correlation between the percentage of M1 macrophages as well as M1/M2 ratio and teratoma grading (**Figures 6G-I** and **Supplementary Figure 9A**). Interestingly, the infiltrated macrophages were more abundant in DCX-specific and mixed region than in SOX2-specific region, indicating different tumor immune microenvironment among neuroectodermal subtypes (**Figure 6J** and **Supplementary Figure 9B**).

On the other hand, we investigated potential role of other potential infiltrated immune cells in PDTs, which is undetectable in CDTs due to immunodeficiency of the injected mice, such as T and NKT cells. PDT02 has higher proportions of T cells and NK cells than PDT01/PDT03 (**Figure 3A**). Although no pronounced difference of interactions between infiltrated immune cells and other cell types was identified among three PDTs (**Figures 5E, F**), PDT02 exhibited significant increased signature-estimated cytotoxicity and mobility but not exhaustion features of NK and T cell compared to PDT01/PDT03 (**Supplementary Figures 10A-C**), thus may contribute to the better prognosis of PDT02.

## Discussion

Pathological similarities were noticed between patient derived immature teratomas and stem cell lines derived immature teratomas, suggesting systematic comparison of PDT and CDT may contribute to understanding of both multi-lineage developmental of stem cell and molecular basis of malignant immature teratomas tumorigenesis/progression. In this study, we conducted the first scRNA-seq analysis on immature ovarian teratomas in adult and investigated the similarities/differences of PDTs and CDTs at single cell level in terms of cell proportion profile, evolutionary trajectory and transcriptional regulatory of the malignant neuroectodermal cells, cell-cell crosstalk and tumor immune microenvironment heterogeneity. Our findings highlight the areas for advances in the biology of immature teratoma that may be useful in the diagnosis and treatment of immature ovarian teratoma.

At single cell resolution, we identified a total of 28 cell clusters, some of which may only contain less than 100 cells. Nevertheless, we systematically conducted comparison of the canonical marker genes with both human fetal cell atlas and annotated stem cell derived teratomas (15, 25), and established the key marker gene list (**Supplementary Table 2**). Such resource may be useful for future bioinformatic and experimental analysis on teratomas to explore the characteristics and clinical relevance of immature teratomas, such as heterogeneity and prognostic cell types. Given the malignant neuroectodermal cells are the well-established cell types for PDT clinically (39), and highly related to prognosis (3), we here in this study mainly focused on these cells as examples to link the pathologic characteristics of PDT to its single cell profile. The malignant neuroectodermal cells can be divided into three clusters based our analysis (i.e., including radial glia, immature neuron, and cycling neural progenitor), and the cluster of radial glia out of 28 clusters presented the specific pathologic markers (e.g., *GFAP* and *SOX2*), thus perfectly reflected the pathologic characteristics of PDTs. Interestingly, these three cell types can be consistently observed in CDTs with the same canonical marker genes (15), indicating CDTs may be used as a model not only for multi-lineage human development, but also for research on immature teratomas, including the tumorigenesis and treatment strategies/drug design. This is further supported by the observation that CDTs and PDTs exhibit a similar evolutionary trajectory. According to our evolutionary trajectory analysis, we observed that radial glia probably evolved from cycling neural progenitors. Interestingly, PDTs from patients with favorable treatment outcomes exhibited evolution inhibition, suggesting the potential important role of tumor cell evolution in tumorigenesis/prognosis of PDTs, which is similar to other cancer types as we described previously (20, 49). Besides radial glia, we noticed the cluster of immature neuron cells as another trajectory end of evolution. This cell type was tagged by a series of markers for early neural development but was rarely mentioned in pathology of immature teratomas (41–44), contributing to the transcriptome disparity/heterogeneity among PDTs and decreasing in proportion in patient with favorable prognosis. Therefore, targeting the overall neural cell progenitor differentiation to promote gliogenesis and inhibit immature neurogenesis may be an alternative strategy for immature teratoma treatment, and the role of immature neuron in PDT is worthy of evaluation in the future.

On the other hand, cell-cell interactions inferred by both CellphoneDB and Cellchat consistently illustrated less prolific interactions of malignant neuroectodermal cells (particularly radial glia) from PDT02 with several types of fibroblasts, such as TGFB1-based interaction in meningeal fibroblast. TGFB1 is required for microglia development (50), and restricted to the choroid plexus and meninges in the developing nervous system (51). Experimentally co-culturing cerebellar slices with meningeal cells can stimulate immature neuron emigration and the formation of radial glial cells (52), while proteins secreted by meninges are involved in the modulation of neuronal and glial cell survival and function (53). Therefore, fibroblast may also play a role in immature teratomas through interacting with the malignant neuroectodermal cells, which is similar to CAFs in other cancer types (19, 54, 55).

Finally, we characterized the tumor immune microenvironment (TIME), which is a major contributing factor to tumor metastasis, relapse, and drug resistance (56, 57). In teratomas, the immune cells may be derived from differentiation of teratomas progenitor cells or host’s immune system infiltration (58). According to scRNA-seq data from CDTs, immune cells differentiated from teratomas progenitor cells seems to be small in number and limited to myeloid cells. In contrast, infiltrated myeloid and T cells exhibited the functional trend with proportions of malignant cell, including MHC-II-mediated antigen presentation and T cytotoxicity. These results suggested that infiltrating immune cells might exert a major influence in TIME of immature teratomas.

Several limitations of this study should be noticed: 1) Due to the rarity of PDTs, the cohort size for scRNA-seq and mIF staining is small, thus the potential correlations of PDT characteristics (e.g., proportions, prolific interactions) with prognosis should be verified in large patient cohorts. 2) We mainly focused on the malignant neuroectodermal cells in this study due to the well-established pathologic evaluation on immature teratomas. However, other cell types out of all 28 cell clusters may also play a role in the diagnosis/prognosis of PDTs through differentiation dysfunction and cell-cell crosstalk (e.g., the PDT-specific clusters of stem cell like/embryonic stem cell and the most prolific interactions between meningeal fibroblast and myofibroblast), which is worthy of further investigation with large sample size. 3) Given ovarian teratomas are typically detected as a large, heterogeneous mass (1), a piece of resected tissue may fail to provide a good representation of entire teratomas, thus need a multi-region validation in the future.

In conclusion, we conducted the first systematic investigation on adult immature ovarian teratomas and provided its comprehensive profile at single cell level, which is a resource for future research on PDT. The comparison of PDTs and CDTs also highlighted the potential usage of CDTs as a model to test drug response/treatment strategy for PDT in the future.

## Data availability statement

The original contributions presented in the study are publicly available. The accession number for the scRNA-seq data of CDTs used in this study can be found at GEO: GSE156170. The remaining data presented in the study are deposited in the gsa-human repository (59), accession number: HRA003823.

## Ethics statement

This study was approved by the Institutional Review Board of West China Second University Hospital (IRB No. 2020112). The patients/participants provided their written informed consent to participate in this study.

## Author contributions

Conception and design: HX, XL, and YS. Administrative support: WW and HX. Collection of study materials or patient samples: XL. Sample preparation and experimental conduction: YuD, JW and QH and YL. Collection and assembly of data: MC and YiD. Data analysis and interpretation: MC, CY, ZW, HF, ZR, and XX. Funding acquisition: HX and YS. All authors contributed to the article and approved the submitted version.

## Supplementary material

Supplementary Figure 1 Clinical performance and pathologic evaluation of three PTC samples.

Supplementary Figure 2 Molecular features of PDT and single cell profile of CDT. **(A)** Normalized expression of selected cell type-specific markers across all clusters in UMAP. **(B)** Correlation of the average expression of each PDT cell type with that of related cell type in human fetal cell altas. **(C)** UMAP visualization of cell types identified from scRNA-seq of the H1-derived CDTs.

Supplementary Figure 3 Characteristics of neuroectodermal cell types in CDT. **(A)** The proportion of cells origin from three germ layers across three PDT samples. **(B)** Left: UMAP presentation of three main neuroectodermal cell types in CDT; Right: expression levels of their representative markers. **(C)** mIF with antibodies against DCX (green) and SOX2 (red). **(D)** Radial glia and cycling neural progenitor transcriptome heterogeneity illustrated by PCA projection across all teratomas. **(E)** The correlation between PCA similarity distance to ter7 and radial glia/cycling neural progenitor proportions in H1-derived CDTs. **(F)** Correlation between average optical density (AOD) of DCX immunofluorescence and grading of immature ovarian teratoma. **(G)** Top: Radial glia proportion across all samples in PDT and CDT; Bottom: expression heatmap of canonical radial glia markers in radial glia cells. **(H)** Top: Proportion of cycling neural progenitor across all samples in PDT and CDT; Bottom: expression heatmap levels of canonical markers of cycling neural progenitor in neural progenitors. **(I)** The correlation between gene expression level and immature neuron proportions with linear and categorical model.

Supplementary Figure 4 Evolution of neuroectodermal cells in CDTs. **(A)** Monocle trajectory of three main neuroectodermal cell types, root was defined by state with minimum CytoTRACE score (less differentiated). **(B)** The proportion of three main neuroectodermal cell types across three monocle trajectory states.

Supplementary Figure 5 Specificity score-based rank for SCENIC regulons across all cell types

Supplementary Figure 6 Interactions among cell types of PDTs. **(A)** CellphoneDB-based cell-cell interaction counts across cell types in three PDT samples. **(B)** The inferred PDT02 vs. PDT01/03 differential number of interactions between cycling neural progenitor/immature neuron and other cells.

Supplementary Figure 7 Copy number variation patterns across all PDT samples with scRNA-seq

Supplementary Figure 8 Pathway enriched in PDT02 among non-immune cells. **(A)** DEG enrichment of non-immune cells between PDT02 and PDT01/03. **(B)** Top: immature neuron proportion across CDT samples; Bottom: the expression heatmap of MHC-II components in CDT-derived myeloid cells.

Supplementary Figure 9 Multiplexed immunofluorescence analysis of PDT. **(A)** mIF staining across all grades of PDTs with antibodies to CD163 (red) and CD68 (yellow). **(B)** mIF staining across DCX-/SOX2-specific and mixed regions with antibodies to DCX (green), SOX2 (red) and CD68 (yellow).

Supplementary Figure 10 Different signature-based functional T cells scores among PDTs. **(A)** Signature-based T cell cytotoxic and effector T cell traffic score of T cells from PDT02 and PDT01/03. **(B)** Signature-based T cell cytotoxic and effector T cell traffic score of NKT cells from PDT02 and PDT01/03. **(C)** Signature-based T cell exhaustion score of T and NKT cells from PDT02 and PDT01/03.

## References

[1] Glick Y Stanislavsky A Yahya Baba Daniel J Bell Rohit Sharma Andrew Murphy Bruno Di Muzio. Immature ovarian teratoma. In: Radiopaediaorg. Radiopaedia.org (2011).

[2] Snir OL DeJoseph M Wong S Buza N Hui P. Frequent homozygosity in both mature and immature ovarian teratomas: A shared genetic basis of tumorigenesis. Modern Pathol an Off J United States Can Acad Pathology Inc (2017) 30(10):1467–75. doi: 10.1038/modpathol.2017.66 28664933

[3] Norris HJ Zirkin HJ Benson WL. Immature (Malignant) teratoma of the ovary: A clinical and pathologic study of 58 cases. Cancer (1976) 37(5):2359–72. doi: 10.1002/1097-0142(197605)37:5<2359::aid-cncr2820370528>3.0.co;2-q 1260722

[4] Djordjevic B Mirkovic J. Germ cell neoplasms of the ovary. Gynecol Pathol (2020), 707–47. doi: 10.1016/B978-0-323-35909-2.00016-3

[5] Bergamini A Sarwar N Ferrandina G Scarfone G Short D Aguiar X. Can we replace adjuvant chemotherapy with surveillance for stage ia-c immature ovarian teratomas of any grade? an international multicenter analysis. Eur J Cancer (Oxford Engl 1990) (2020) 137:136–43. doi: 10.1016/j.ejca.2020.06.033 32763784

[6] Albanell J Bosl GJ Reuter VE Engelhardt M Franco S Moore MA. Telomerase activity in germ cell cancers and mature teratomas. J Natl Cancer Inst (1999) 91(15):1321–6. doi: 10.1093/jnci/91.15.1321 10433622

[7] Heskett MB Sanborn JZ Boniface C Goode B Chapman J Garg K. Multiregion exome sequencing of ovarian immature teratomas reveals 2n near-diploid genomes, paucity of somatic mutations, and extensive allelic imbalances shared across mature, immature, and disseminated components. Modern Pathol (2020) 33(6):1193–206. doi: 10.1038/s41379-019-0446-y PMC7286805 31911616

[8] Van Nieuwenhuysen E Busschaert P Neven P Han SN Moerman P Liontos M. The genetic landscape of 87 ovarian germ cell tumors. Gynecol Oncol (2018) 151(1):61–8. doi: 10.1016/j.ygyno.2018.08.013 30170975

[9] Chen J Li Y Wu J Liu Y Kang S. Whole-exome sequencing reveals potential germline and somatic mutations in 60 malignant ovarian germ cell tumors†. Biol Reprod (2021) 105(1):164–78. doi: 10.1093/biolre/ioab052 33739378

[10] Ye CJ Zhan Y Yang R Li Y Dong R. Single-cell transcriptional profiling identifies a cluster of potential metastasis-associated Ube2c+ cells in immature ovarian teratoma. Biochem Biophys Res Commun (2020) 528(3):567–73. doi: 10.1016/j.bbrc.2020.05.144 32505346

[11] Willis RA. The structure of teratomata. J Pathol (1935) 40(1):1–36. doi: 10.1002/path.1700400102

[12] Thurlbeck WM Scully RE. Solid teratoma of the ovary. a clinicopathological analysis of 9 cases. Cancer (1960) 13:804–11. doi: 10.1002/1097-0142(196007/08)13:4<804::aid-cncr2820130423>3.0.co;2-v 13838271

[13] Böcker W. [Who classification of breast tumors and tumors of the female genital organs: Pathology and genetics]. Verh Dtsch Ges Pathol (2002) 86:116–9. 12647359

[14] Lensch MW Schlaeger TM Zon LI Daley GQ. Teratoma formation assays with human embryonic stem cells: A rationale for one type of human-animal chimera. Cell Stem Cell (2007) 1(3):253–8. doi: 10.1016/j.stem.2007.07.019 18371359

[15] McDonald D Wu Y Dailamy A Tat J Parekh U Zhao D. Defining the teratoma as a model for multi-lineage human development. Cell (2020) 183(5):1402–19.e18. doi: 10.1016/j.cell.2020.10.018 33152263 PMC7704916

[16] Parekh U McDonald D Dailamy A Wu Y Cordes T Zhang K. Charting oncogenicity of genes and variants across lineages *Via* multiplexed screens in teratomas. iScience (2021) 24(10):103149. doi: 10.1016/j.isci.2021.103149 34646987 PMC8496177

[17] WHO classification of tumours editorial board. Female genital tumours (2020) 5th ed. 2020 (Lyon: International agency for research on cancer).

[18] Liao X Xia X Su W Yan H Ma Y Xu L. Association of recurrent Apobec3b alterations with the prognosis of gastric-type cervical adenocarcinoma. Gynecol Oncol (2022) 165(1):105–13. doi: 10.1016/j.ygyno.2022.01.036 35151492

[19] Luo H Xia X Huang LB An H Cao M Kim GD. Pan-cancer single-cell analysis reveals the heterogeneity and plasticity of cancer-associated fibroblasts in the tumor microenvironment. Nat Commun (2022) 13(1):6619. doi: 10.1038/s41467-022-34395-2 36333338 PMC9636408

[20] Luo H Xia X Kim GD Liu Y Xue Z Zhang L. Characterizing dedifferentiation of thyroid cancer by integrated analysis. Sci Adv (2021) 7(31):eabf3657. doi: 10.1126/sciadv.abf3657 PMC8318367 34321197

[21] Zheng GXY Terry JM Belgrader P Ryvkin P Bent ZW Wilson R. Massively parallel digital transcriptional profiling of single cells. Nat Commun (2017) 8:14049. doi: 10.1038/ncomms14049 28091601 PMC5241818

[22] Butler A Hoffman P Smibert P Papalexi E Satija R. Integrating single-cell transcriptomic data across different conditions, technologies, and species. Nat Biotechnol (2018) 36(5):411–20. doi: 10.1038/nbt.4096 PMC6700744 29608179

[23] Wolock SL Lopez R Klein AM. Scrublet: Computational identification of cell doublets in single-cell transcriptomic data. Cell Syst (2019) 8(4):281–91.e9. doi: 10.1016/j.cels.2018.11.005 30954476 PMC6625319

[24] Hafemeister C Satija R. Normalization and variance stabilization of single-cell rna-seq data using regularized negative binomial regression. Genome Biol (2019) 20(1):296. doi: 10.1186/s13059-019-1874-1 31870423 PMC6927181

[25] Cao J O'Day DR Pliner HA Kingsley PD Deng M Daza RM. A human cell atlas of fetal gene expression. Science (2020) 370(6518):eaba7721. doi: 10.1126/science.aba7721 PMC7780123 33184181

[26] Liu B Li C Li Z Wang D Ren X Zhang Z. An entropy-based metric for assessing the purity of single cell populations. Nat Commun (2020) 11(1):3155. doi: 10.1038/s41467-020-16904-3 32572028 PMC7308400

[27] Trapnell C Cacchiarelli D Grimsby J Pokharel P Li S Morse M. The dynamics and regulators of cell fate decisions are revealed by pseudotemporal ordering of single cells. Nat Biotechnol (2014) 32(4):381–6. doi: 10.1038/nbt.2859 PMC4122333 24658644

[28] Gulati GS Sikandar SS Wesche DJ Manjunath A Bharadwaj A Berger MJ. Single-cell transcriptional diversity is a hallmark of developmental potential. Science (2020) 367(6476):405–11. doi: 10.1126/science.aax0249 PMC7694873 31974247

[29] Aibar S Gonzalez-Blas CB Moerman T Huynh-Thu VA Imrichova H Hulselmans G. Scenic: Single-cell regulatory network inference and clustering. Nat Methods (2017) 14(11):1083–6. doi: 10.1038/nmeth.4463 PMC5937676 28991892

[30] Cheng S Li Z Gao R Xing B Gao Y Yang Y. A pan-cancer single-cell transcriptional atlas of tumor infiltrating myeloid cells. Cell (2021) 184(3):792–809.e23. doi: 10.1016/j.cell.2021.01.010 33545035

[31] Bagaev A Kotlov N Nomie K Svekolkin V Gafurov A Isaeva O. Conserved pan-cancer microenvironment subtypes predict response to immunotherapy. Cancer Cell (2021) 39(6):845–65.e7. doi: 10.1016/j.ccell.2021.04.014 34019806

[32] Liu X Jin S Hu S Li R Pan H Liu Y. Single-cell transcriptomics links malignant T cells to the tumor immune landscape in cutaneous T cell lymphoma. Nat Commun (2022) 13(1):1158. doi: 10.1038/s41467-022-28799-3 35241665 PMC8894386

[33] Szabo PA Levitin HM Miron M Snyder ME Senda T Yuan J. Single-cell transcriptomics of human T cells reveals tissue and activation signatures in health and disease. Nat Commun (2019) 10(1):4706. doi: 10.1038/s41467-019-12464-3 31624246 PMC6797728

[34] Efremova M Vento-Tormo M Teichmann SA Vento-Tormo R. Cellphonedb: Inferring cell-cell communication from combined expression of multi-subunit ligand-receptor complexes. Nat Protoc (2020) 15(4):1484–506. doi: 10.1038/s41596-020-0292-x 32103204

[35] Jin S Guerrero-Juarez CF Zhang L Chang I Ramos R Kuan C-H. Inference and analysis of cell-cell communication using cellchat. Nat Commun (2021) 12(1):1088. doi: 10.1038/s41467-021-21246-9 33597522 PMC7889871

[36] La Manno G Soldatov R Zeisel A Braun E Hochgerner H Petukhov V. Rna velocity of single cells. Nature (2018) 560(7719):494–8. doi: 10.1038/s41586-018-0414-6 PMC6130801 30089906

[37] Bergen V Lange M Peidli S Wolf FA Theis FJ. Generalizing rna velocity to transient cell states through dynamical modeling. Nat Biotechnol (2020) 38(12):1408–14. doi: 10.1038/s41587-020-0591-3 32747759

[38] Bergers G Song S. The role of pericytes in blood-vessel formation and maintenance. Neuro Oncol (2005) 7(4):452–64. doi: 10.1215/S1152851705000232 PMC1871727 16212810

[39] Siegelman ES. Mri of the female pelvis. Body Mri Elsevier (2005) . p:269–342. doi: 10.1016/B978-0-7216-3740-2.50012-1

[40] Fang X Zheng P Tang J Liu Y. Cd24: From a to z. Cell Mol Immunol (2010) 7(2):100–3. doi: 10.1038/cmi.2009.119 PMC4001892 20154703

[41] Mu L Berti L Masserdotti G Covic M Michaelidis TM Doberauer K. Soxc transcription factors are required for neuronal differentiation in adult hippocampal neurogenesis. J Neurosci (2012) 32(9):3067–80. doi: 10.1523/JNEUROSCI.4679-11.2012 PMC3356877 22378879

[42] Miller JA Nathanson J Franjic D Shim S Dalley RA Shapouri S. Conserved molecular signatures of neurogenesis in the hippocampal subgranular zone of rodents and primates. Development (2013) 140(22):4633–44. doi: 10.1242/dev.097212 PMC3817946 24154525

[43] Tortosa E Galjart N Avila J Sayas CL. Map1b regulates microtubule dynamics by sequestering Eb1/3 in the cytosol of developing neuronal cells. EMBO J (2013) 32(9):1293–306. doi: 10.1038/emboj.2013.76 PMC3642684 23572079

[44] Wang W Wang M Yang M Zeng B Qiu W Ma Q. Transcriptome dynamics of hippocampal neurogenesis in macaques across the lifespan and aged humans. Cell Res (2022) 32(8):729–43. doi: 10.1038/s41422-022-00678-y PMC9343414 35750757

[45] Zhou Q Anderson DJ. The bhlh transcription factors Olig2 and Olig1 couple neuronal and glial subtype specification. Cell (2002) 109(1):61–73. doi: 10.1016/s0092-8674(02)00677-3 11955447

[46] Murdoch JN Eddleston J Leblond-Bourget N Stanier P Copp AJ. Sequence and expression analysis of Nhlh1: A basic helix-Loop-Helix gene implicated in neurogenesis. Dev Genet (1999) 24(1-2):165–77. doi: 10.1002/(SICI)1520-6408(1999)24:1/2<165::AID-DVG15>3.0.CO;2-V 10079519

[47] Skaggs K Martin DM Novitch BG. Regulation of spinal interneuron development by the olig-related protein Bhlhb5 and notch signaling. Development (2011) 138(15):3199–211. doi: 10.1242/dev.057281 PMC3133912 21750031

[48] Chen T Wang X Guo L Wu M Duan Z Lv J. Embryonic stem cells promoting macrophage survival and function are crucial for teratoma development. Front Immunol (2014) 5:275. doi: 10.3389/fimmu.2014.00275 25071759 PMC4082241

[49] Chen HN Shu Y Liao F Liao X Zhang H Qin Y. Genomic evolution and diverse models of systemic metastases in colorectal cancer. Gut (2022) 71(2):322–32. doi: 10.1136/gutjnl-2020-323703 PMC8762014 33632712

[50] Butovsky O Jedrychowski MP Moore CS Cialic R Lanser AJ Gabriely G. Identification of a unique tgf-β-dependent molecular and functional signature in microglia. Nat Neurosci (2014) 17(1):131–43. doi: 10.1038/nn.3599 PMC4066672 24316888

[51] Krieglstein K Strelau J Schober A Sullivan A Unsicker K. Tgf-beta and the regulation of neuron survival and death. J Physiol Paris (2002) 96(1-2):25–30. doi: 10.1016/s0928-4257(01)00077-8 11755780

[52] Hartmann D Schulze M Sievers J. Meningeal cells stimulate and direct the migration of cerebellar external granule cells in vitro. J Neurocytol (1998) 27(6):395–409. doi: 10.1023/a:1006998609999 10192521

[53] Ishikawa K Ohe Y Tatemoto K. Synthesis and secretion of insulin-like growth factor (Igf)-ii and igf binding protein-2 by cultivated brain meningeal cells. Brain Res (1995) 697(1-2):122–9. doi: 10.1016/0006-8993(95)00798-U 8593568

[54] Franco OE Shaw AK Strand DW Hayward SW. Cancer associated fibroblasts in cancer pathogenesis. Semin Cell Dev Biol (2010) 21(1):33–9. doi: 10.1016/j.semcdb.2009.10.010 PMC2823834 19896548

[55] Sahai E Astsaturov I Cukierman E DeNardo DG Egeblad M Evans RM. A framework for advancing our understanding of cancer-associated fibroblasts. Nat Rev Cancer (2020) 20(3):174–86. doi: 10.1038/s41568-019-0238-1 PMC7046529 31980749

[56] Binnewies M Roberts EW Kersten K Chan V Fearon DF Merad M. Understanding the tumor immune microenvironment (Time) for effective therapy. Nat Med (2018) 24(5):541–50. doi: 10.1038/s41591-018-0014-x PMC5998822 29686425

[57] Huang T-X Fu L. The immune landscape of esophageal cancer. Cancer Commun (Lond) (2019) 39(1):79. doi: 10.1186/s40880-019-0427-z 31771653 PMC6878621

[58] Karlsson KR Cowley S Martinez FO Shaw M Minger SL James W. Homogeneous monocytes and macrophages from human embryonic stem cells following coculture-free differentiation in m-csf and il-3. Exp Hematol (2008) 36(9):1167–75. doi: 10.1016/j.exphem.2008.04.009 PMC2635571 18550257

[59] Database Resources of the National Genomics Data Center. China National center for bioinformation in 2022. Nucleic Acids Res (2022) 50(D1):D27–d38. doi: 10.1093/nar/gkab951 34718731 PMC8728233

