# Supplementary material for: Characterization of immature ovarian teratomas through single-cell transcriptome
Source: Front Immunol. 2023 Mar 3;14:1131814. doi: 10.3389/fimmu.2023.1131814 (PMC10020330; doi:10.3389/fimmu.2023.1131814)
Supplement: Supplementary Figure 1 — Clinical performance and pathologic evaluation of three PTC samples. [file DataSheet_2.pdf]

Figure S1

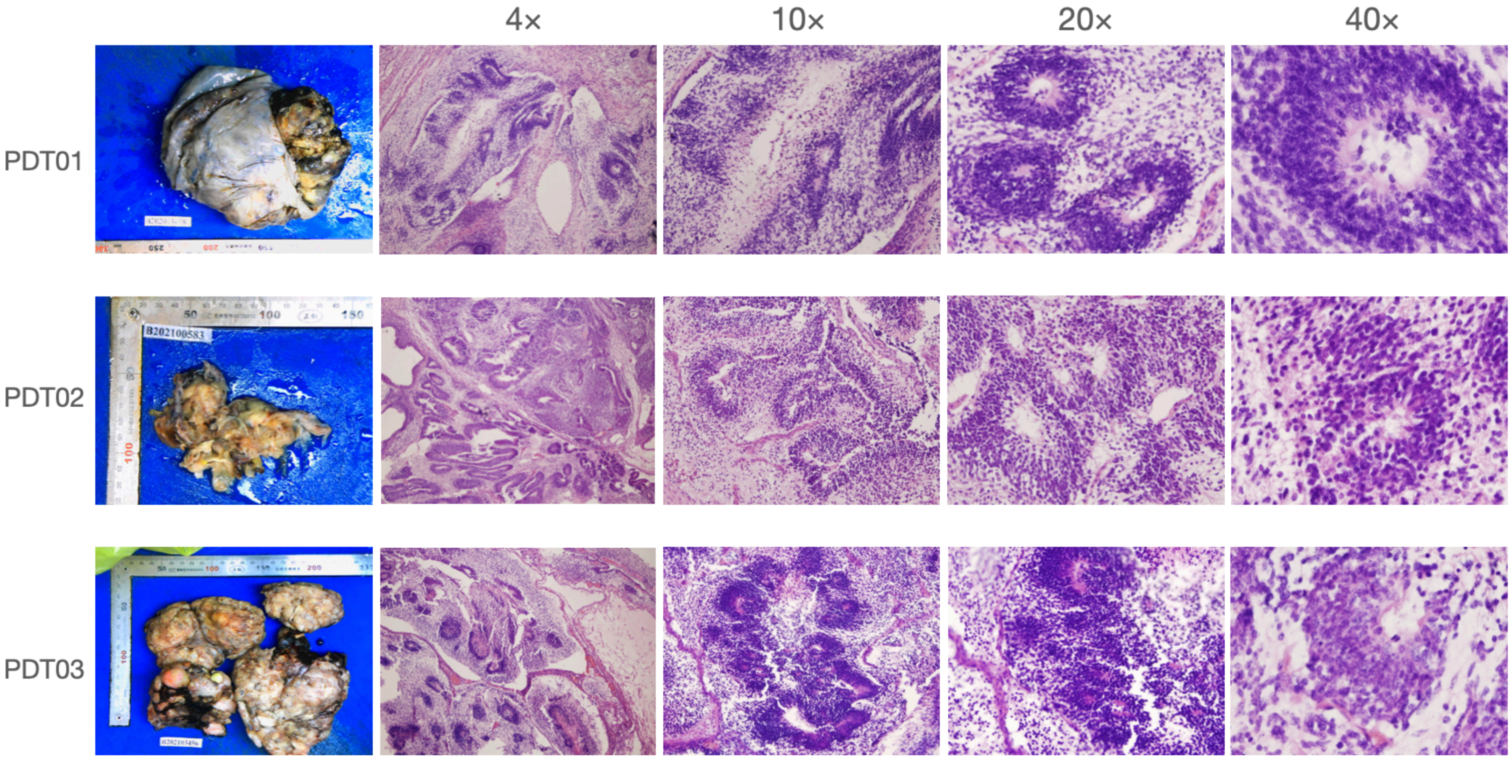

Figure S2

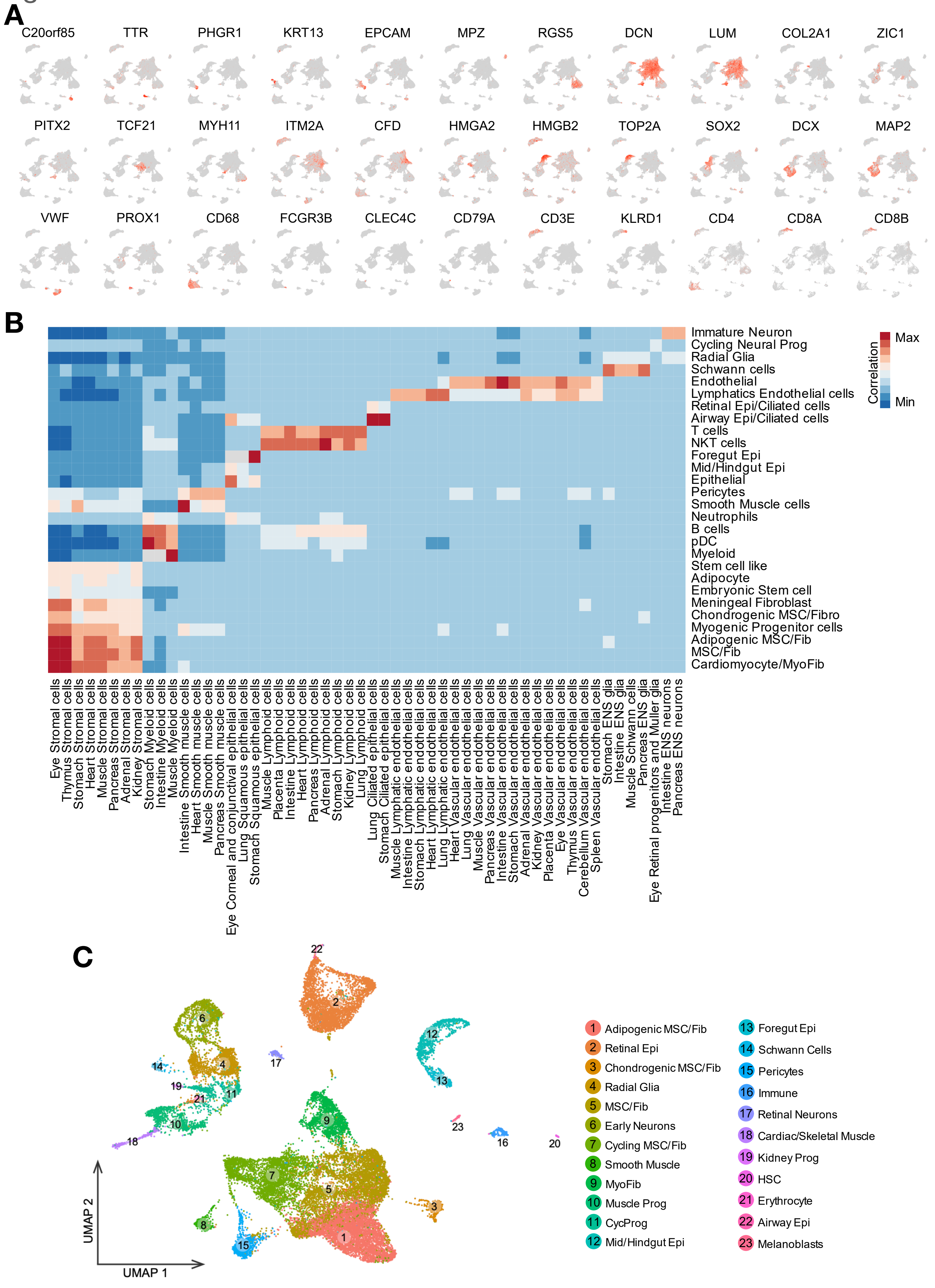
A
C20orf85
TTR
PHGR1
KRT13
EPCAM
MPZ
RGS5
DCN
LUM
COL2A1
ZIC1
PITX2
TCF21
MYH11
ITM2A
CFD
HMGA2
HMGB2
TOP2A
SOX2
DCX
MAP2
VWF
PROX1
CD68
FCGR3B
CLEC4C
CD79A
CD3E
KLRD1
CD4
CD8A
CD8B
B
Immature Neuron
Cycling Neural Prog
Radial Glia
Schwann cells
Endothelial
Lymphatics Endothelial cells
Retinal Epi/Ciliated cells
Airway Epi/Ciliated cells
T cells
NKT cells
Foregut Epi
Mid/Hindgut Epi
Epithelial
Pericytes
Smooth Muscle cells
Neutrophils
B cells
pDC
Myeloid
Stem cell like
Adipocyte
Embryonic Stem cell
Meningeal Fibroblast
Chondrogenic MSC/Fibro
Myogenic Progenitor cells
Adipogenic MSC/Fib
MSC/Fib
Cardiomyocyte/MyoFib
Correlation
Max
Min
Eye Stromal cells
Thymus Stromal cells
Stomach Stromal cells
Heart Stromal cells
Muscle Stromal cells
Pancreas Stromal cells
Adrenal Stromal cells
Kidney Stromal cells
Stomach Myeloid cells
Intestine Myeloid cells
Muscle Myeloid cells
Intestine Smooth muscle cells
Heart Smooth muscle cells
Muscle Smooth muscle cells
Pancreas Smooth muscle cells
Eye Corneal and conjunctival epithelial cells
Lung Squamous epithelial cells
Stomach Squamous epithelial cells
Muscle Lymphoid cells
Placenta Lymphoid cells
Intestine Lymphoid cells
Heart Lymphoid cells
Pancreas Lymphoid cells
Adrenal Lymphoid cells
Stomach Lymphoid cells
Kidney Lymphoid cells
Lung Lymphoid cells
Lung Ciliated epithelial cells
Stomach Ciliated epithelial cells
Muscle Lymphatic endothelial cells
Intestine Lymphatic endothelial cells
Stomach Lymphatic endothelial cells
Heart Lymphatic endothelial cells
Lung Lymphatic endothelial cells
Heart Vascular endothelial cells
Lung Vascular endothelial cells
Muscle Vascular endothelial cells
Pancreas Vascular endothelial cells
Intestine Vascular endothelial cells
Stomach Vascular endothelial cells
Adrenal Vascular endothelial cells
Kidney Vascular endothelial cells
Placenta Vascular endothelial cells
Eye Vascular endothelial cells
Thymus Vascular endothelial cells
Cerebellum Vascular endothelial cells
Spleen Vascular endothelial cells
Stomach ENS glia
Intestine ENS glia
Muscle Schwann cells
Pancreas ENS glia
Eye Retinal progenitors and Muller glia
Intestine ENS neurons
Pancreas ENS neurons
C
UMAP 2
UMAP 1
1 Adipogenic MSC/Fib
2 Retinal Epi
3 Chondrogenic MSC/Fib
4 Radial Glia
5 MSC/Fib
6 Early Neurons
7 Cycling MSC/Fib
8 Smooth Muscle
9 MyoFib
10 Muscle Prog
11 CycProg
12 Mid/Hindgut Epi
13 Foregut Epi
14 Schwann Cells
15 Pericytes
16 Immune
17 Retinal Neurons
18 Cardiac/Skeletal Muscle
19 Kidney Prog
20 HSC
21 Erythrocyte
22 Airway Epi
23 Melanoblasts

Figure S3

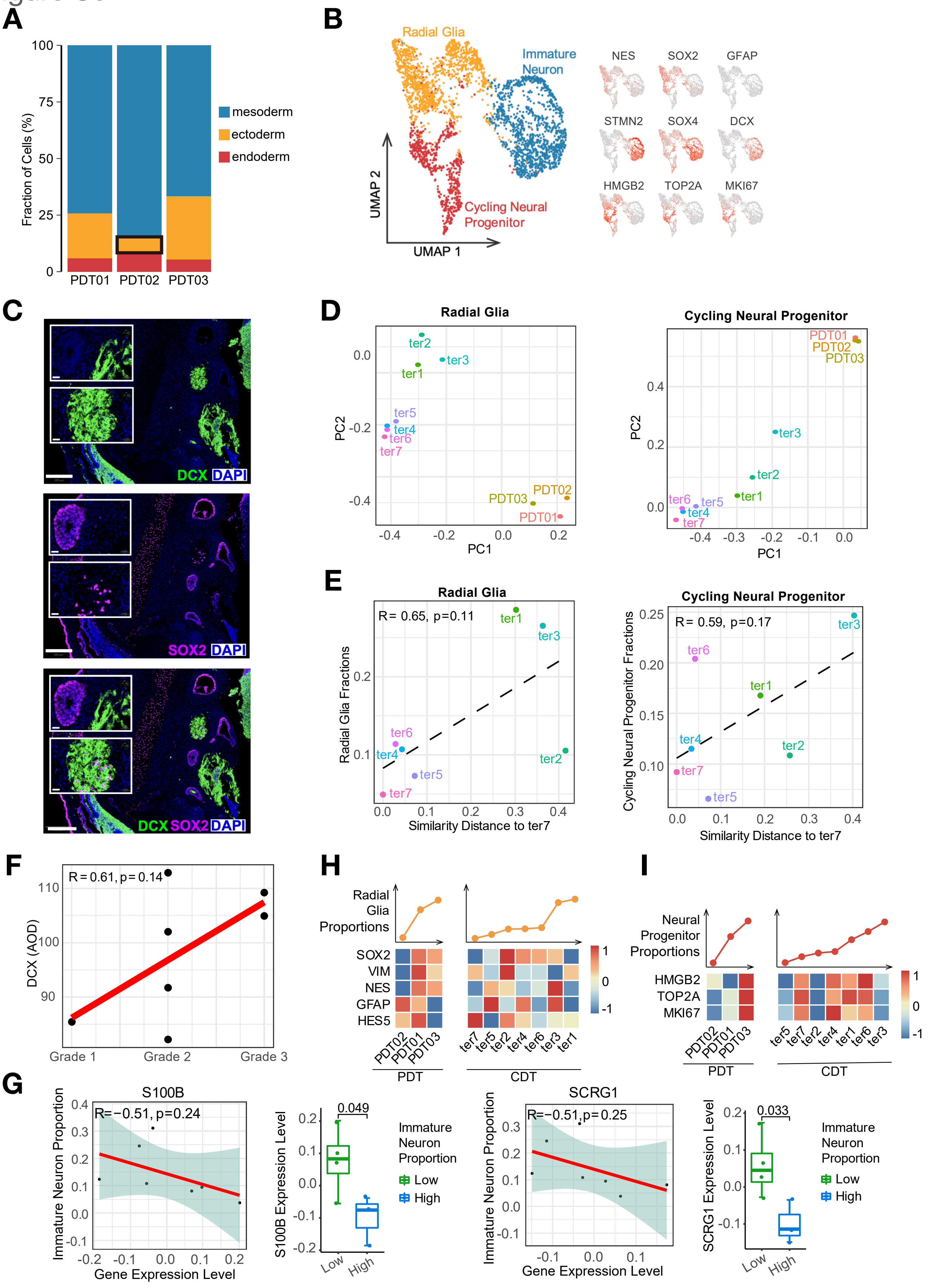

A
B
C
D
E
F
G
H
I
Fraction of Cells (%)
mesoderm
ectoderm
endoderm
PDT01
PDT02
PDT03
Radial Glia
Immature Neuron
Cycling Neural Progenitor
UMAP 1
UMAP 2
NES
SOX2
GFAP
STMN2
SOX4
DCX
HMGB2
TOP2A
MKI67
DCX DAPI
SOX2 DAPI
DCX SOX2 DAPI
Radial Glia
Cycling Neural Progenitor
PC1
PC2
R= 0.65, p=0.11
R= 0.59, p=0.17
Radial Glia Fractions
Cycling Neural Progenitor Fractions
Similarity Distance to ter7
R = 0.61, p = 0.14
DCX (AOD)
Grade 1
Grade 2
Grade 3
Radial Glia Proportions
Neural Progenitor Proportions
SOX2
VIM
NES
GFAP
HES5
PDT
CDT
S100B
SCRG1
R=−0.51, p=0.24
R=−0.51, p=0.25
Immature Neuron Proportion
Gene Expression Level
S100B Expression Level
SCRG1 Expression Level
0.049
0.033
Low
High

Figure S4

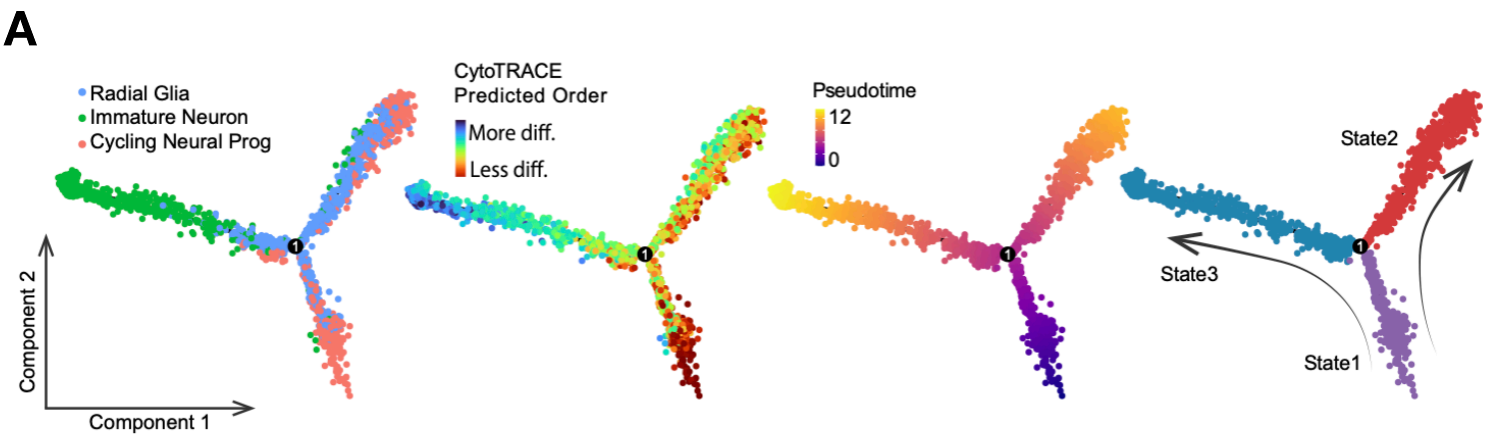
A
Radial Glia
Immature Neuron
Cycling Neural Prog
Component 2
Component 1
CytoTRACE
Predicted Order
More diff.
Less diff.
Pseudotime
12
0
State2
State3
State1
1

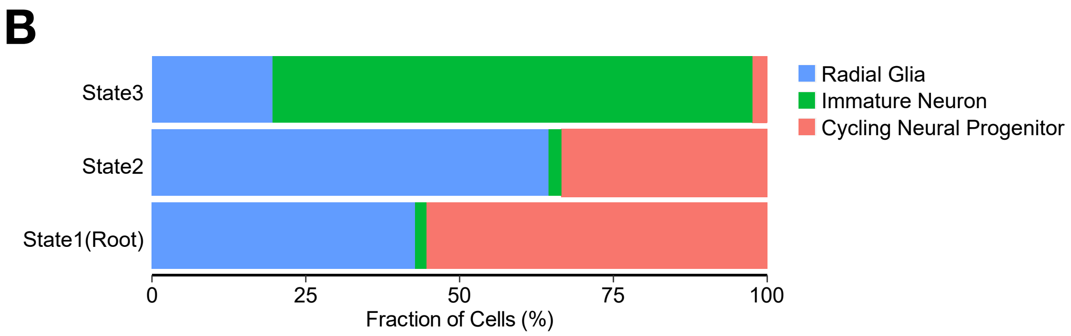
B
State3
State2
State1(Root)
0
25
50
75
100
Fraction of Cells (%)
Radial Glia
Immature Neuron
Cycling Neural Progenitor

Figure S5

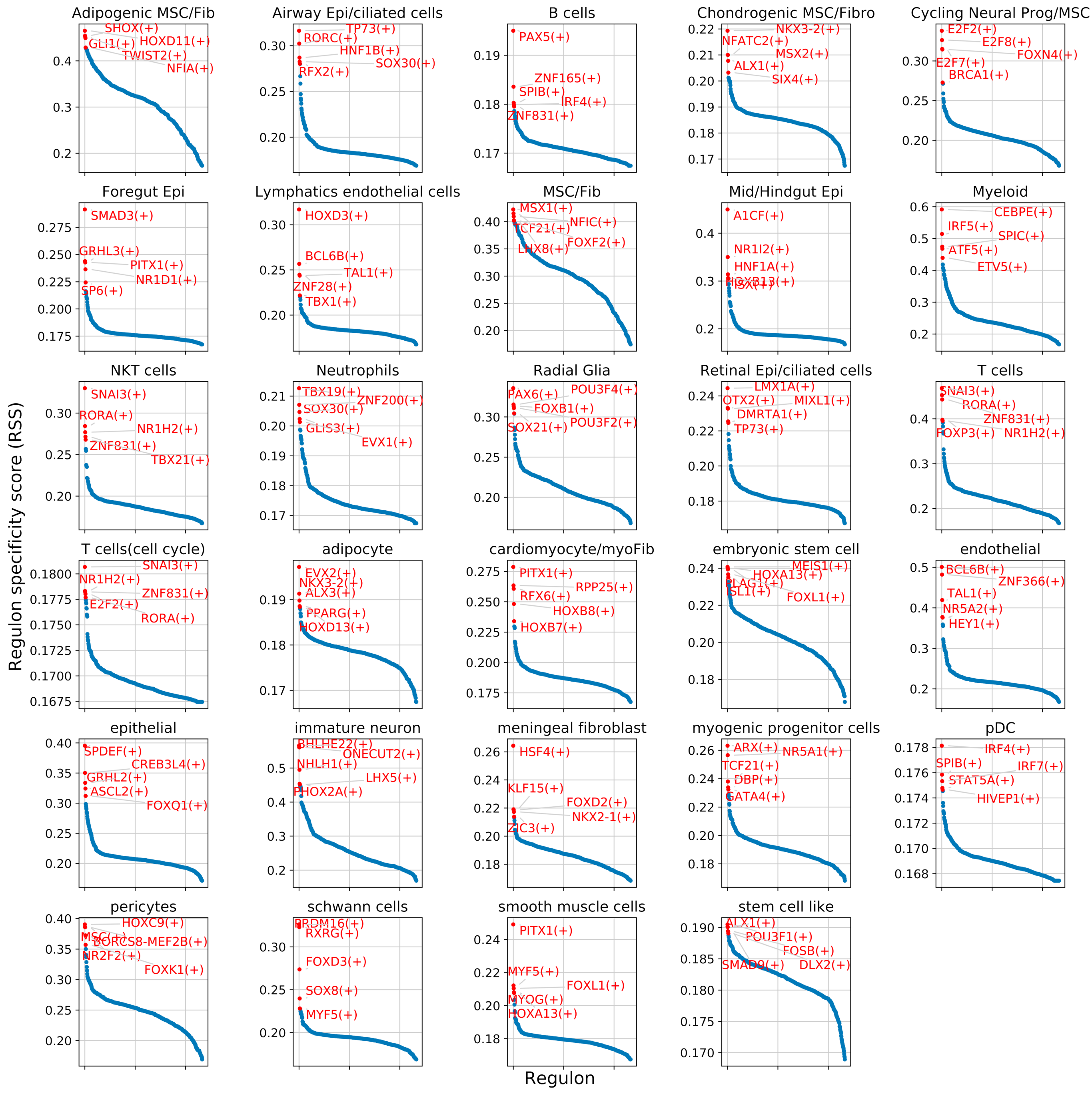
Adipogenic MSC/Fib
Airway Epi/ciliated cells
B cells
Chondrogenic MSC/Fibro
Cycling Neural Prog/MSC
Foregut Epi
Lymphatics endothelial cells
MSC/Fib
Mid/Hindgut Epi
Myeloid
NKT cells
Neutrophils
Radial Glia
Retinal Epi/ciliated cells
T cells
T cells(cell cycle)
adipocyte
cardiomyocyte/myoFib
embryonic stem cell
endothelial
epithelial
immature neuron
meningeal fibroblast
myogenic progenitor cells
pDC
pericytes
schwann cells
smooth muscle cells
stem cell like
Regulon specificity score (RSS)
Regulon

Figure S6

A

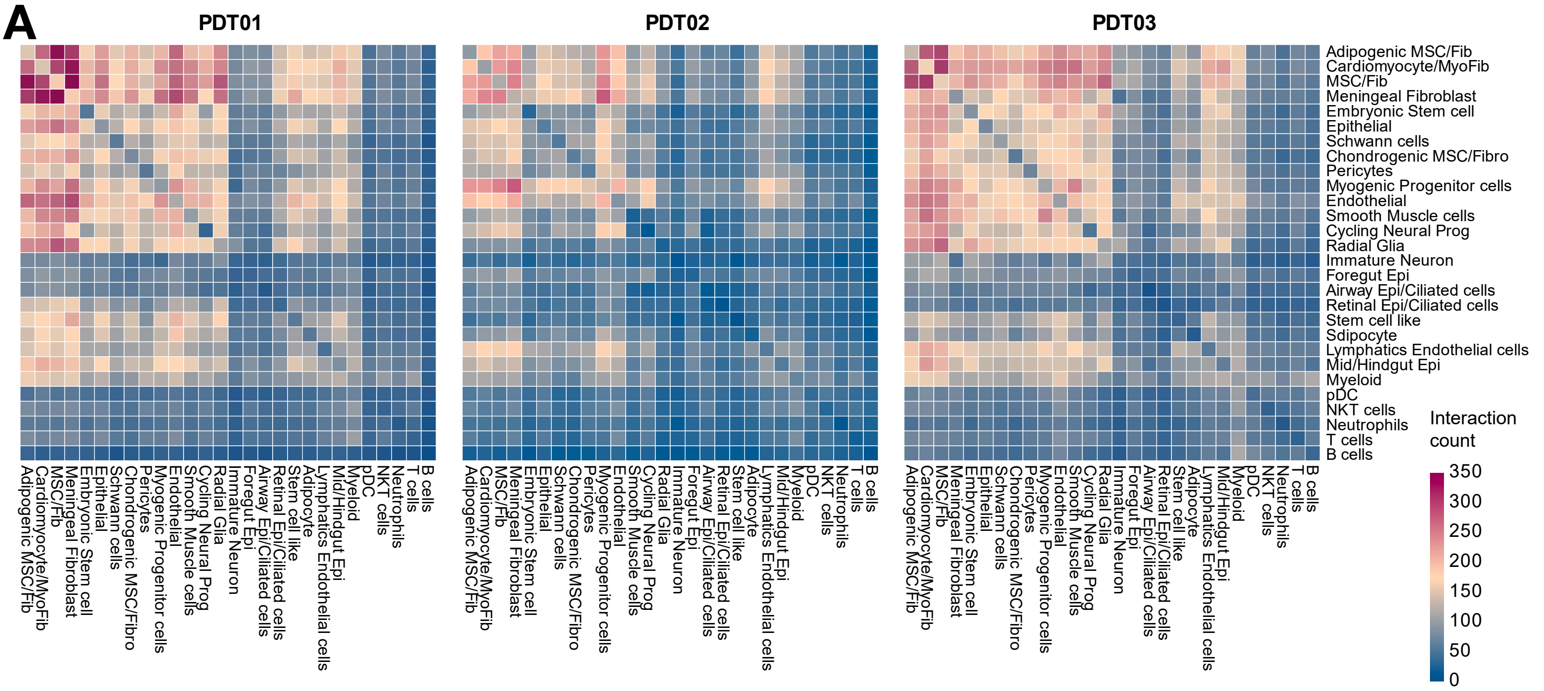

PDT01
PDT02
PDT03
Adipogenic MSC/Fib
Cardiomyocyte/MyoFib
MSC/Fib
Meningeal Fibroblast
Embryonic Stem cell
Epithelial
Schwann cells
Chondrogenic MSC/Fibro
Pericytes
Myogenic Progenitor cells
Endothelial
Smooth Muscle cells
Cycling Neural Prog
Radial Glia
Immature Neuron
Foregut Epi
Airway Epi/Ciliated cells
Retinal Epi/Ciliated cells
Stem cell like
Sdipocyte
Lymphatics Endothelial cells
Mid/Hindgut Epi
Myeloid
pDC
NKT cells
Neutrophils
T cells
B cells
Interaction count
350
300
250
200
150
100
50
0

B

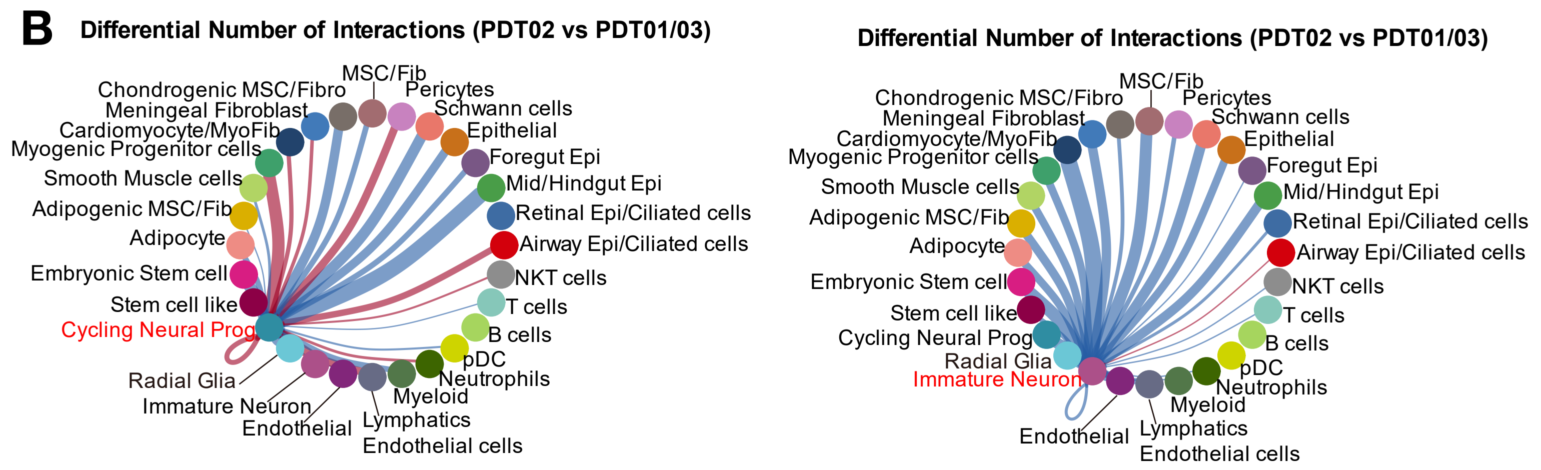

Differential Number of Interactions (PDT02 vs PDT01/03)
Differential Number of Interactions (PDT02 vs PDT01/03)
MSC/Fib
Chondrogenic MSC/Fibro
Pericytes
Meningeal Fibroblast
Schwann cells
Cardiomyocyte/MyoFib
Epithelial
Myogenic Progenitor cells
Foregut Epi
Smooth Muscle cells
Mid/Hindgut Epi
Adipogenic MSC/Fib
Retinal Epi/Ciliated cells
Adipocyte
Airway Epi/Ciliated cells
Embryonic Stem cell
NKT cells
Stem cell like
T cells
Cycling Neural Prog
B cells
pDC
Radial Glia
Neutrophils
Immature Neuron
Myeloid
Endothelial
Lymphatics
Endothelial cells

Figure S7

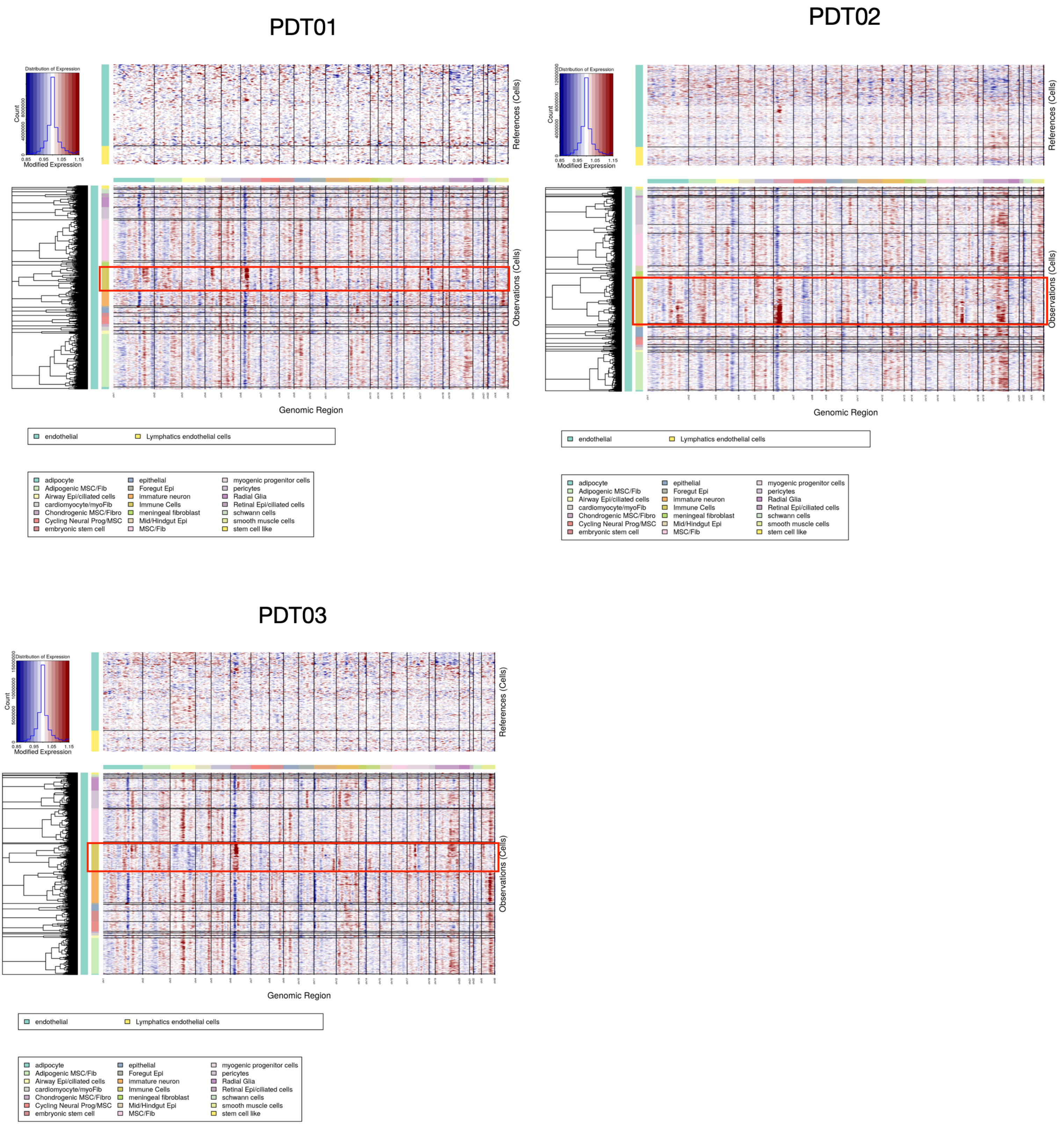
PDT01
PDT02
PDT03
Distribution of Expression
Count
Modified Expression
References (Cells)
Observations (Cells)
Genomic Region
endothelial
Lymphatics endothelial cells
adipocyte
Adipogenic MSC/Fib
Airway Epi/ciliated cells
cardiomyocyte/myoFib
Chondrogenic MSC/Fibro
Cycling Neural Prog/MSC
embryonic stem cell
epithelial
Foregut Epi
immature neuron
Immune Cells
meningeal fibroblast
Mid/Hindgut Epi
MSC/Fib
myogenic progenitor cells
pericytes
Radial Glia
Retinal Epi/ciliated cells
schwann cells
smooth muscle cells
stem cell like

Figure S8

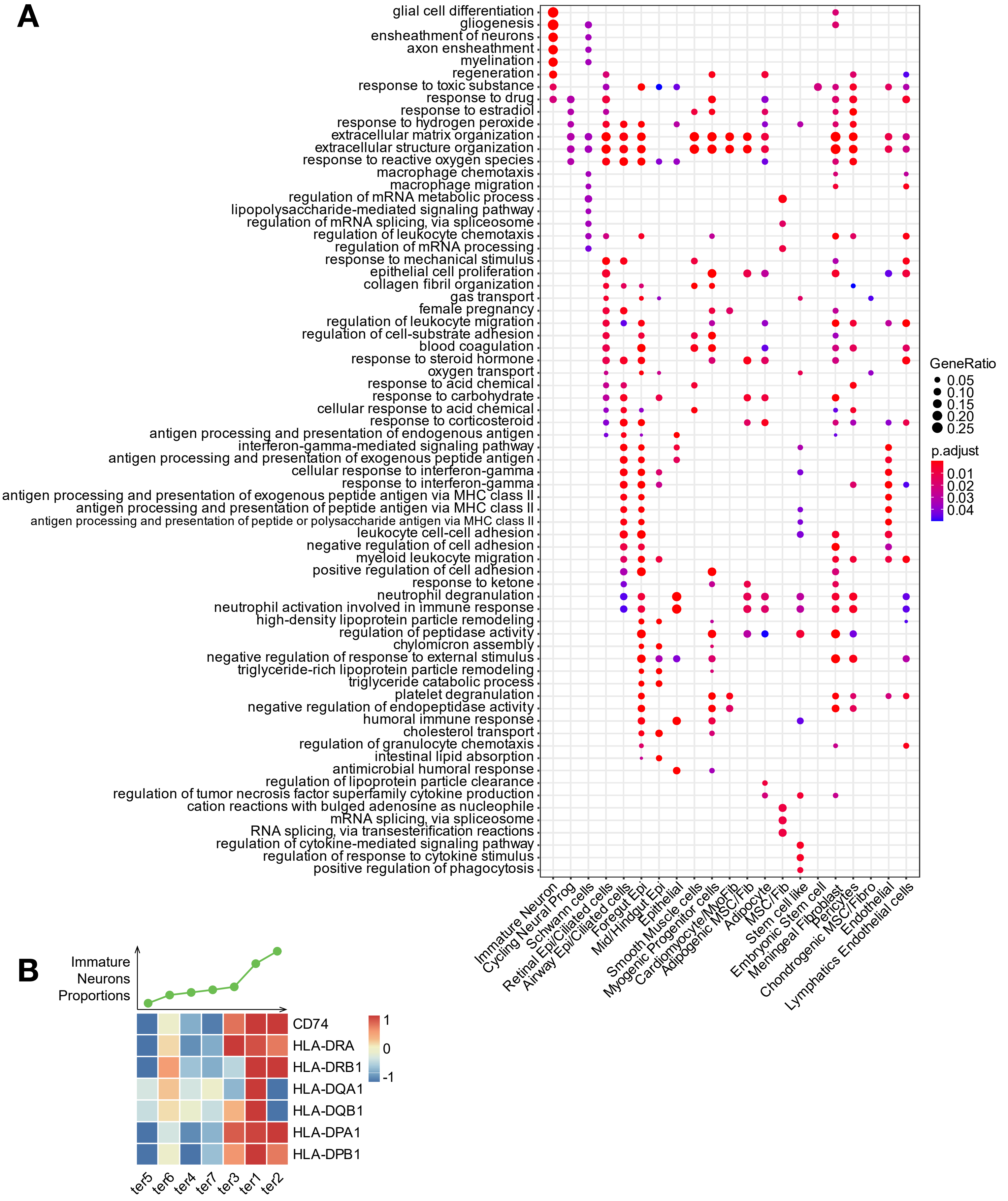

A
glial cell differentiation
gliogenesis
ensheathment of neurons
axon ensheathment
myelination
regeneration
response to toxic substance
response to drug
response to estradiol
response to hydrogen peroxide
extracellular matrix organization
extracellular structure organization
response to reactive oxygen species
macrophage chemotaxis
macrophage migration
regulation of mRNA metabolic process
lipopolysaccharide-mediated signaling pathway
regulation of mRNA splicing, via spliceosome
regulation of leukocyte chemotaxis
regulation of mRNA processing
response to mechanical stimulus
epithelial cell proliferation
collagen fibril organization
gas transport
female pregnancy
regulation of leukocyte migration
regulation of cell-substrate adhesion
blood coagulation
response to steroid hormone
oxygen transport
response to acid chemical
response to carbohydrate
cellular response to acid chemical
response to corticosteroid
antigen processing and presentation of endogenous antigen
interferon-gamma-mediated signaling pathway
antigen processing and presentation of exogenous peptide antigen
cellular response to interferon-gamma
response to interferon-gamma
antigen processing and presentation of exogenous peptide antigen via MHC class II
antigen processing and presentation of peptide antigen via MHC class II
antigen processing and presentation of peptide or polysaccharide antigen via MHC class II
leukocyte cell-cell adhesion
negative regulation of cell adhesion
myeloid leukocyte migration
positive regulation of cell adhesion
response to ketone
neutrophil degranulation
neutrophil activation involved in immune response
high-density lipoprotein particle remodeling
regulation of peptidase activity
chylomicron assembly
negative regulation of response to external stimulus
triglyceride-rich lipoprotein particle remodeling
triglyceride catabolic process
platelet degranulation
negative regulation of endopeptidase activity
humoral immune response
cholesterol transport
regulation of granulocyte chemotaxis
intestinal lipid absorption
antimicrobial humoral response
regulation of lipoprotein particle clearance
regulation of tumor necrosis factor superfamily cytokine production
cation reactions with bulged adenosine as nucleophile
mRNA splicing, via spliceosome
RNA splicing, via transesterification reactions
regulation of cytokine-mediated signaling pathway
regulation of response to cytokine stimulus
positive regulation of phagocytosis
Immature Neuron
Cycling Neural Prog
Schwann cells
Retinal Epi/Ciliated cells
Airway Epi/Ciliated cells
Foregut Epi
Mid/Hindgut Epi
Epithelial
Smooth Muscle cells
Myogenic Progenitor cells
Cardiomyocyte/MyoFib
Adipogenic MSC/Fib
Adipocyte
MSC/Fib
Stem cell like
Embryonic Stem cell
Meningeal Fibroblast
Pericytes
Chondrogenic MSC/Fibro
Endothelial
Lymphatics Endothelial cells
GeneRatio
0.05
0.10
0.15
0.20
0.25
p.adjust
0.01
0.02
0.03
0.04
B
Immature Neurons Proportions
CD74
HLA-DRA
HLA-DRB1
HLA-DQA1
HLA-DQB1
HLA-DPA1
HLA-DPB1
1
0
-1
ter5
ter6
ter4
ter7
ter3
ter1
ter2

Figure S9

A

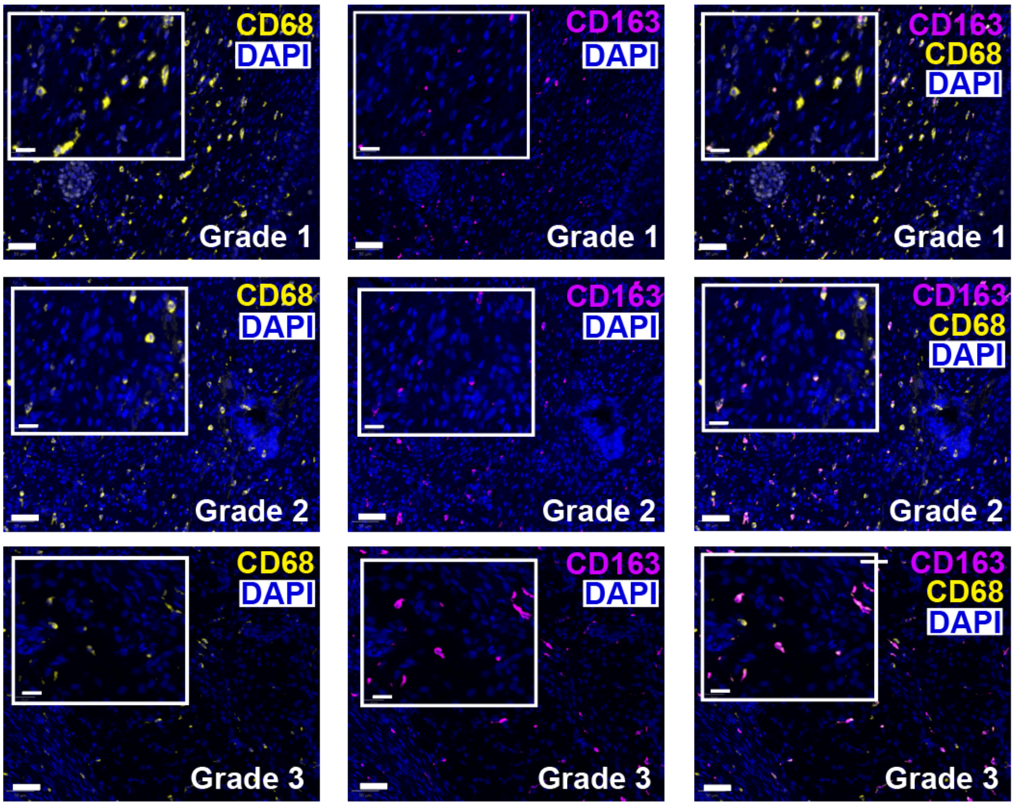
CD68
DAPI
Grade 1
CD163
DAPI
Grade 1
CD163
CD68
DAPI
Grade 1
CD68
DAPI
Grade 2
CD163
DAPI
Grade 2
CD163
CD68
DAPI
Grade 2
CD68
DAPI
Grade 3
CD163
DAPI
Grade 3
CD163
CD68
DAPI
Grade 3

B

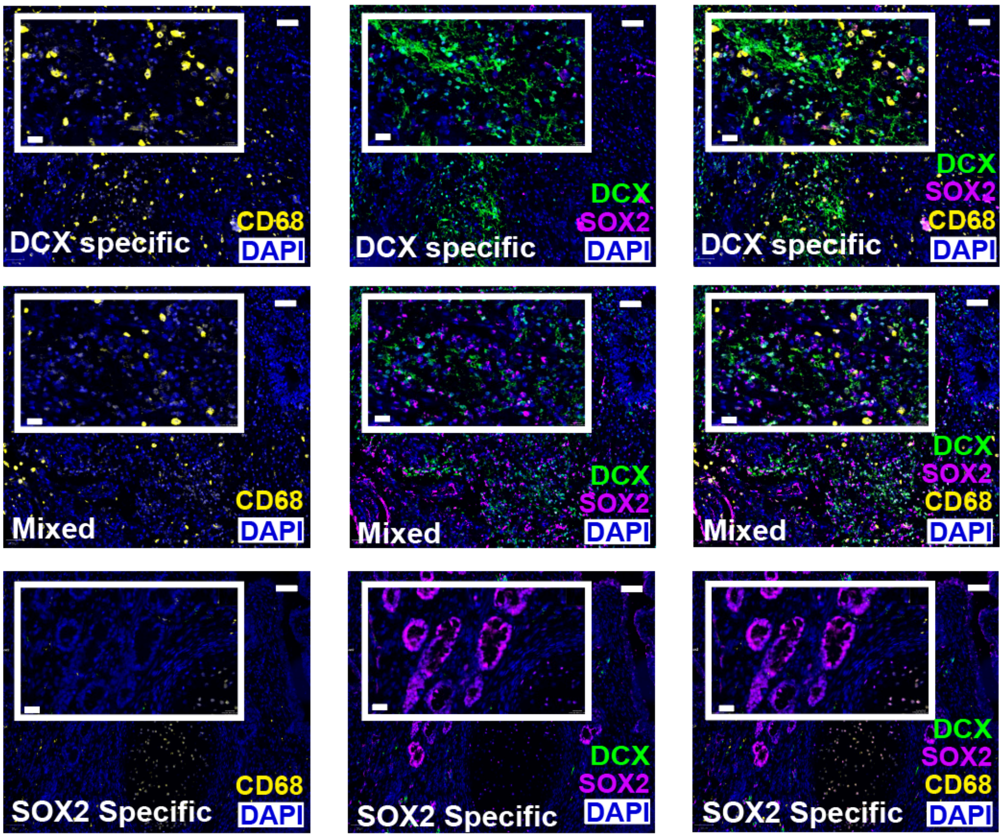
DCX specific
CD68
DAPI
DCX specific
DCX
SOX2
DAPI
DCX specific
DCX
SOX2
CD68
DAPI
Mixed
CD68
DAPI
Mixed
DCX
SOX2
DAPI
Mixed
DCX
SOX2
CD68
DAPI
SOX2 Specific
CD68
DAPI
SOX2 Specific
DCX
SOX2
DAPI
SOX2 Specific
DCX
SOX2
CD68
DAPI

Figure S10

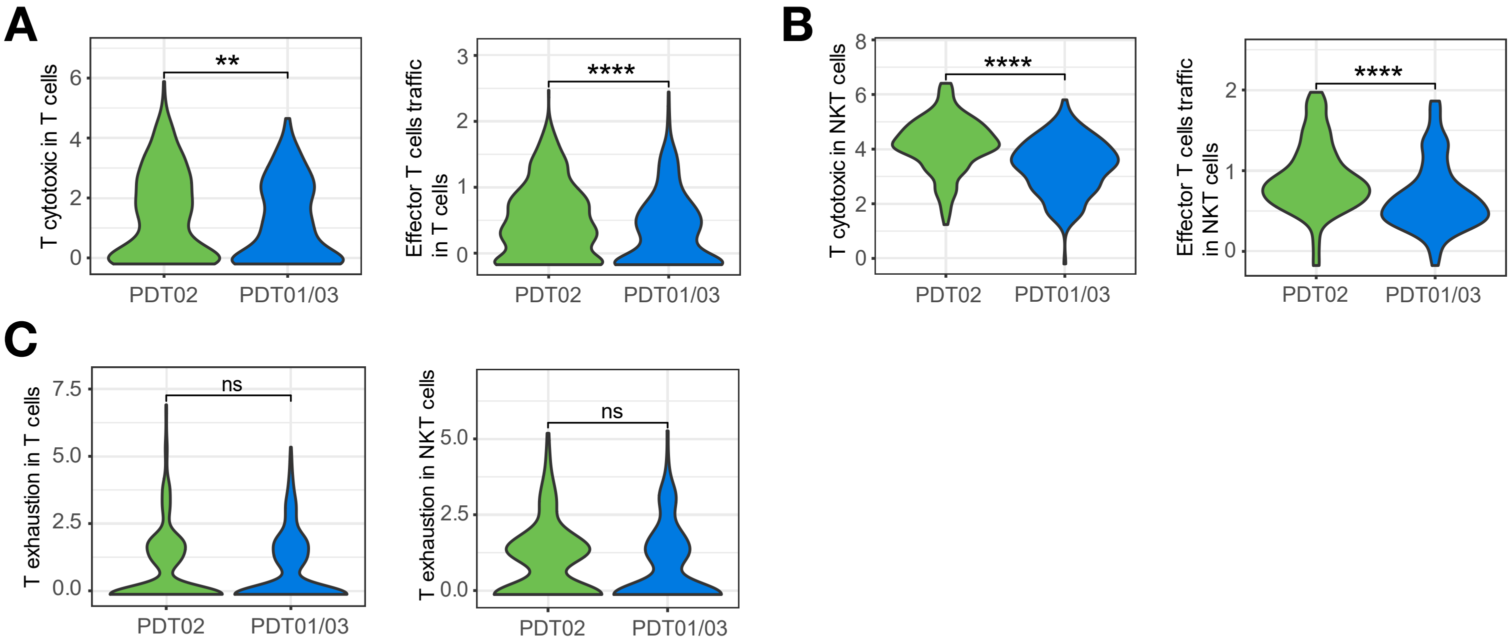
A
T cytotoxic in T cells
**
PDT02
PDT01/03
Effector T cells traffic in T cells
****
B
T cytotoxic in NKT cells
****
Effector T cells traffic in NKT cells
****
C
T exhaustion in T cells
ns
T exhaustion in NKT cells
ns
